# Increased water temperature contributes to a chondrogenesis response in the eyes of spotted wolffish

**DOI:** 10.1038/s41598-024-63370-8

**Published:** 2024-05-31

**Authors:** Rebecca R. Kwabiah, Eva Weiland, Sarah Henderson, Ignacio Vasquez, Hélène Paradis, Denise Tucker, Iliana Dimitrov, Danielle Gardiner, Stephanie Tucker, Nicholas Newhook, Danny Boyce, Giuseppe Scapigliati, Simon Kirby, Javier Santander, Robert L. Gendron

**Affiliations:** 1 Division of Biomedical Sciences, Faculty of Medicine, Memorial University, St. John’s, NL A1B 3V6 Canada; 2 Marine Microbial Pathogenesis and Vaccinology Lab, Department of Ocean Sciences, Memorial University, St. John’s, NL A1C 5S7 Canada; 3 Dr. Joe Brown Aquatic Research Building (JBARB), Department of Ocean Sciences, Memorial University, St. John’s, NL A1C 5S7 Canada; 4 Università della Tuscia, Dipartimento DIBAF, Viterbo, Italy; 5 Discipline of Laboratory Medicine, Faculty of Medicine, Memorial University, St. John’s, NL A1B 3V6 Canada; 6 Present Address: Faculty of Biotechnology, Mannheim University of Applied Sciences, Paul-Wittsack-Straße 10, 68163 Mannheim, Germany

**Keywords:** Marine biology, Cell growth, Cartilage development, Projection and prediction

## Abstract

Adult vertebrate cartilage is usually quiescent. Some vertebrates possess ocular scleral skeletons composed of cartilage or bone. The morphological characteristics of the spotted wolffish (*Anarhichas minor*) scleral skeleton have not been described. Here we assessed the scleral skeletons of cultured spotted wolffish, a globally threatened marine species. The healthy spotted wolffish we assessed had scleral skeletons with a low percentage of cells staining for the chondrogenesis marker sex-determining region Y-box (Sox) 9, but harboured a population of intraocular cells that co-express immunoglobulin M (IgM) and Sox9. Scleral skeletons of spotted wolffish with grossly observable eye abnormalities displayed a high degree of perochondrial activation as evidenced by cellular morphology and expression of proliferating cell nuclear antigen (PCNA) and phosphotyrosine. Cells staining for cluster of differentiation (CD) 45 and IgM accumulated around sites of active chondrogenesis, which contained cells that strongly expressed Sox9. The level of scleral chondrogenesis and the numbers of scleral cartilage PCNA positive cells increased with the temperature of the water in which spotted wolffish were cultured. Our results provide new knowledge of differing Sox9 spatial tissue expression patterns during chondrogenesis in normal control and ocular insult paradigms. Our work also provides evidence that spotted wolffish possess an inherent scleral chondrogenesis response that may be sensitive to temperature. This work also advances the fundamental knowledge of teleost ocular skeletal systems.

## Introduction

Many marine teleosts are visually guided predators that rely on visual acuity for feeding and survival. Understanding unique anatomical and functional features of the visual systems of North Atlantic teleosts could reveal the significance of these systems to the health and survival of both wild and cultured animals. We have recently characterized the visual system of the North Atlantic lumpfish (*Cyclopterus lumpus*)^1^. The characteristics we have observed in the eyes of this species and their importance as models of ocular and immune disorders^2–7^ has prompted comparative analyses toward exploring the visual systems of other locally available North Atlantic marine teleosts as described in the work herein on spotted wolffish.

The three species of wolffish (Northern, Spotted and Atlantic) inhabiting eastern Canadian marine waters have declined over the past 20 years and are protected under the Canada Strategy Species at Risk Act (SARA)^8^. While the Atlantic Wolffish is listed as “special concern”, the Northern and Spotted wolffish (*Anarhichas minor*) are now listed as “threatened”^8–10^. Threats to wolffish include bycatch mortality in commercial fisheries and disturbance of marine habitat^9^. Spotted wolffish are commonly found at depths of 100–400 m in water temperatures that can be less than 5 °C but their thermal range in the wild can be broader including temperatures from 2 to 8  C or higher^10,11^. They can also secrete low levels of antifreeze proteins into their plasma^12^. Observations of wild spotted wolffish are scarce and most knowledge has been gained from fisheries data, hatchery observations, or inferrences from Atlantic wolffish (*Anarhichas lupus*) observations. Spotted wolffish have been cultured for industrial purposes since the 1980s^11–14^. Relevant to its threatened status, the culture of spotted wolffish could also serve as a means for protecting the species or understanding how environmental conditions might impact the health of these animals toward better protection in the wild. Given the challenges of observing these fish in the wild and although they are constrained in their movement in culture compared to migrating to their thermal preference in the wild, herein we have focused on cultured fish.

Existing literature indicates several environmental conditions such as temperature^15^, salinity^16^ and water oxygenation levels^17^ impacting the general health of spotted wolffish in a culture setting. One report describes ocular abnormalities including cataract, lens extrusion and severe inflammation associated with changes in water temperature and salinity in spotted wolffish^18^. Another study describes xanthomatosis and nephrocalcinosis in aquarium-housed spotted wolffish^19^. Persistent nodavirus infection has been observed in spotted wolffish larva^20,21^. To our knowledge there are no studies that describe the anatomy, histology and histopathology of the adult wolffish eye or the host response to any physical ocular insult in spotted wolffish.

Spotted wolffish ocular tissues arise and become pigmented relatively early in development^22^. While there are few studies of the ocular immune responses of wolffish, these animals do have humoral immune parameters^22,23^. Spotted wolffish larvae are well developed upon hatching and show adaptive immune responses shortly post hatch^23,24^. In culture, spotted wolffish possess a low susceptibility to viral and bacterial pathogens, with only one bacterial disease (infection by atypical *Aeromonas* sp.) reported under stressful conditions^14,20^. Our previous work has described the morphological and molecular changes in ocular tissues of lumpfish experimentally challenged intraperitoneally with the marine pathogen *V. anguillarum*^4,6^ but studies of the response of spotted wolffish eyes to exogenous insults have not been reported.

Scleral skeletons, which are present in eyes of most animals except humans, primates and rodents, are thought to provide structural support and integrity to the eye globe, particularly in animals that move fast or swim to deep depths where hydrostatic pressures are high^25^. Previous work has shown that ocular retinal pigment epithelium regulates the development of scleral cartilage^26^. Recent work in teleosts provides evidence that scleral cartilage induction through chondrogenesis originates at the ora serrata, which is generally known as the point of transition from the ciliary epithelium to the neural retina^27,28^. Consistent positioning of scleral cartilage relative to these tissues suggests that the mechanism for cartilage induction in teleosts is spatially conserved between species. However, differences in scleral cartilage appearance between species suggest temporal variation^27^.

Chondrogenesis is a key developmental process required for skeletal and cartilage formation as well as for repair and regeneration in skeletal tissue damage. Mature cartilage is formed sequentially from mesenchymal chondroblasts in inner perichondrial layers that underly cartilage matrix secreted by matured chondrocytes that migrate and differentiate from the perichondrial chondroblasts. The differential histological stain safranin O marks both the early and mature cartilage matrix and can be used to visualize the stage at which isogenous groups of maturing chondrocytes start producing de novo islands of cartilage matrix which then coalesce to form consolidated volumes of mature cartilage^29^. While there is a body of literature describing in vitro models of chondrogenesis, there are gaps in knowledge and lack of resources for in vivo models of chondrogenesis. Robust models with which to study the molecular pathways and cellular biological mechanisms underlying chondrogenesis are lacking. Spontaneous healing of partial-thickness cartilage defects has been observed in infantile and immature rat models^30,31^. However, complete healing is not observed and it is a long term process. Another recent model of chondrogenesis proposed is zebrafish, which display jaw cartilage regeneration in response to ligament transection^32^. Although Marconi et al. (2020) have described adult chondrogenesis and spontaneous repair of cartilage in the skate *Leucoraja erinacea,* to our knowledge, there are no reports describing scleral cartilage chondrogenesis as experimental models for cartilage repair/regeneration^33^. Markers of chondrogenesis such as *sox6*, *sox9*, and *col2a1* are observed in both chondrichythians (cartilaginous fish) and osteichthyians (bony fish), suggesting cartilage development is a highly conserved process^33^. Scleral cartilage contains a unique morphology that incorporates multiple elements of cartilage types^34^. In teleost, hyperthermia can increase the occurrence of vertebral fusion and upregulates the expression of chondrogenesis markers including bone morphogenetic proteins (BMP) 4 and 5, Sox9, Basic helix-loop-helix transcription factor (Twist), and Runx family transcription factor (Runx) 2^35^. Thus, further characterization of scleral cartilage could provide valuable insight into teleost ocular biology.

## Results

### Gross morphological pathology in spotted wolffish eyes

**Figure 1:**
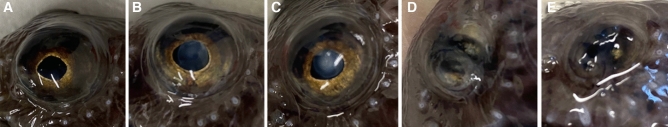
Gross morphology of spotted wolffish presenting with normal versus abnormal eyes. (**A**) Normal; (**B**) Retrolenticular opacity; (**C**) Anterior opacity; (**D**) and (**E**) Ocular deformation and regression, respectively.

The majority of the 979 spotted wolffish cultured in the JBARB facility have largely proportioned proptosed eyes that are considered normal and do not present grossly observable abnormalities or opacities (Fig. 1).

**Figure 2:**
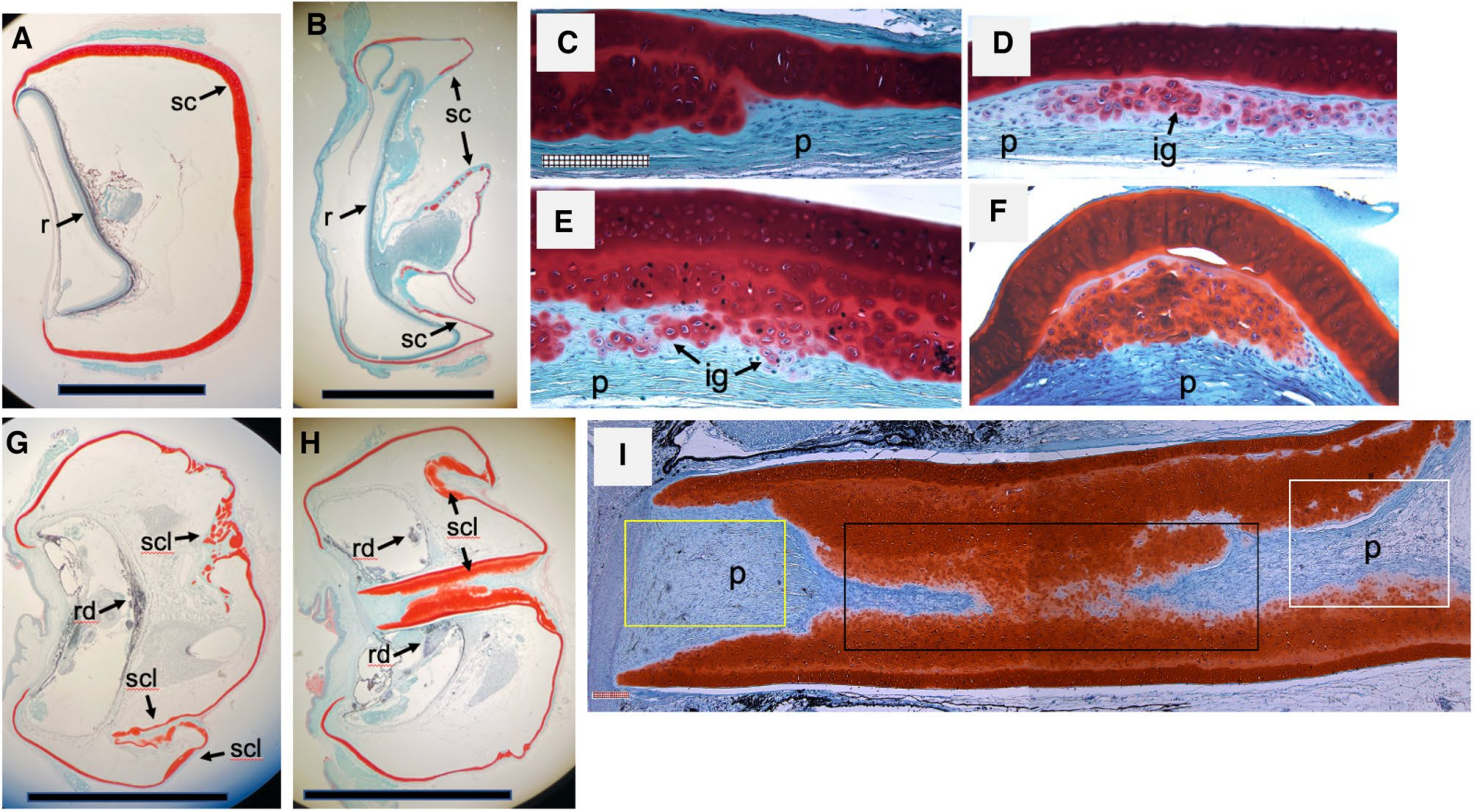
Histological features of spotted wolffish presenting with normal versus abnormal eyes. Whole eye sections were stained with Safranin O-fast green, a red/orange stain marking cartilage matrix. Areas of perichondrium (p) and maturing chondrocytes did not stain with safranin O-fast green. (**A**) Normal adult lumpfish whole eye shown for comparative purposes. (**B**) Normal spotted wolffish whole eye (the eye shown collapsed during paraffin embedding processing as with most spotted wolffish eyes). r, retina; sc, scleral cartilage. (**C**) Higher power view of the scleral cartilage of a spotted wolfish with a normal eye. Whole eye sections showing ocular deformation (**G**) and regression (**H**) associated with scleral cartilage lesion-like (scl) and retinal dysplasia (rd) from two separate spotted wolffish. (**D**), (**E**) and (**F**) Higher magnifications of scleral cartilage lesion-like tissue with isogenous groups of de novo cartilage (ig, arrowed). (**I**) Higher magnification of the eye in (**H**) showing globe-wide scleral cartilage lesion-like tissue traversing the center of the eye from posterior to anterior. Scale bars in (**A**), (**B**), (**G**), (**H**) are 5 milimeters. Whole eye sections in (**A**, **B**, **G**, **H**) are oriented with anterior facing left and posterior facing right. Panels (**C**–**F**) magnification: X200 ; panel I magnification: X5; scale bar subdivisions in (**C**) and (**I**) are 10 µm.

Grossly visible unilateral ocular abnormalities occurring sporadically in a small proportion (approximate frequency of 0.01%) of the colony were observed. These deformities ranged from anterior opacities such as cataract, retrolenticular opacity, exophthalmos with ocular inflammation and hemorrhage and, ocular deformation and regression. Some of these phenotypes are shown in Fig. 1. Most eyes from animals harboring ocular deformation and regression were considerably more firm in consistency than the normal spotted wolfish eyes. Some of the abnormal eyes appeared lobulated or segmented upon enucleation which correlated with histopathological findings (see Fig. 2). One of the eyes of one animal with severe exophthalmos displayed hemorrhage through the cornea upon enucleation.

### Histopathology in spotted wolffish eyes

A differential histochemical analysis was next performed to further explore the anatomical features of the ocular tissues in the cultured spotted wolffish. A safranin O fast green differential histological stain was used to reveal cartilage matrix as a red/orange stain (safranin O) against a light greenish blue counterstain (fast green). Histological analysis showed that normal healthy spotted wolffish possess relatively unremarkable scleral skeletons with a low degree of active chondrogenesis as evidenced by mainly consolidated (i.e., absence of isogenous groups of cartilage secreting chondrocytes) safranin O staining (Fig. 2B,C). Some wolffish presenting with normal eyes displayed a low level of scleral cartilage chondrogenesis. Compared to spotted wolffish, adult lumpfish scleral skeletons were even less remarkable, generally thicker and, as we have noticed over 5 years of histologically assessing both normal and abnormal lumpfish eyes, showed very low levels of discernable de novo chondrogenesis (Fig. 2A). Even at a whole eye globe histological level, eyes of spotted wolffish with grossly observable abnormalities displayed a range of microscopic ocular pathology including extensive general dysplasia, intraocular hemorrhage and various degrees of retinal dysplasia which differed considerably across the individual cases (Fig. 2G,H). A striking characteristic shared by all the individual cases of grossly observable ocular abnormality in these spotted wolffish was the presence of distinct scleral cartilage abnormalities we define here as “lesion-like” areas of tissue featuring classic cartilage isogenous groups, indicative of separate clusters of de novo chondrogenesis^27^ which are apparent in the cases shown at higher magnifications in Fig. 2D,E,F. The spotted wolffish scleral cartilage histopathology was mildest in the cases of anterior opacity and most severe in the cases of ocular deformation and regression. In one of these animals, the eye appeared to be lobulated and safranin O staining indicated whole eye globe-wide scleral cartilage lesion-like tissue traversing the center of the eye from posterior to anterior (Fig. 2I).

### Detection of chondrogenesis and immune markers in ocular tissues of spotted wolffish eyes

Since the genome of spotted wolffish is not yet characterized, we used a number of approaches to validate a panel of anti-teleost and/or anti-mammalian monoclonal antibody reagents against CD45, Sox9, PCNA, IgM and phosphorylated tyrosine to detect the corresponding protein epitopes in spotted wolffish tissues^1,36–41^.

**Figure 3:**
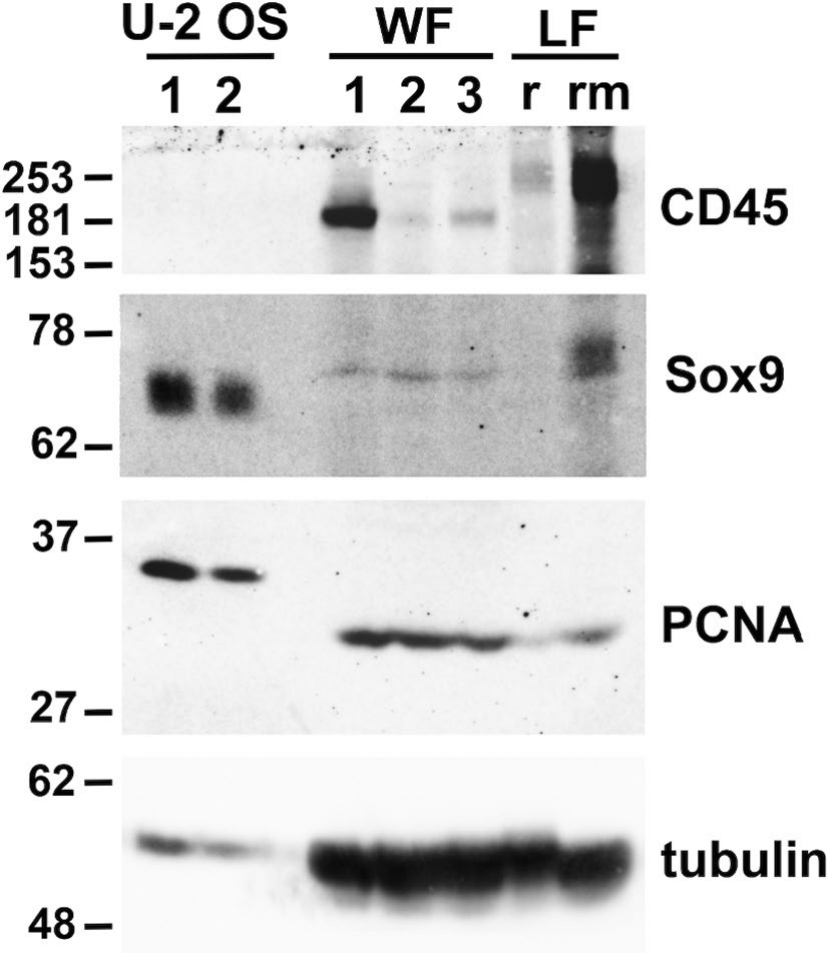
Western blotting of CD45, Sox9 and PCNA in spotted wolffish ocular tissues. U-2 OS human osteosarcoma cell line (lane 1: 5 µg; lane 2: 2.5 µg) was used as a positive control for both Sox9 and PCNA expression, and as a negative control for CD45 expression. Spotted wolffish (WF) retinal tissues: lanes 1, 2 and 3 (200 μg) and Lumpfish (LF) retinal (r: 200 μg) and rete mirabile (rm: 150 μg) tissues were used as positive control for CD45 expression. Antibodies against the above markers detected bands migrating with molecular weights (kDa) similar to the positive control samples (kDa of protein ladder is shown at left) in wolffish retinal tissue combined with rete mirabile tissue in specimens from three different animals (WF: lanes 1, 2 and 3; 200 µg). Tubulin was used as a control for loading and protein preparation integrity.

Western blot analyses for CD45, Sox9 and PCNA, using the same antibody reagents we chose to use for IHC analysis of these proteins, revealed the expression of these markers in spotted wolffish retinal and rete mirabile ocular tissues. The relative molecular weights of most of these markers in spotted wolffish ocular tissues were similar to the positive control tissues or cell line (Fig. 3 and see discussion).

Spotted wolffish IgM purified from blood was detected by Western blotting with the anti-lumpfish IgM antibody, which signal was abrogated by pre-absorption with purified lumpfish IgM (Supplemental Fig. 5).

**Figure 4:**
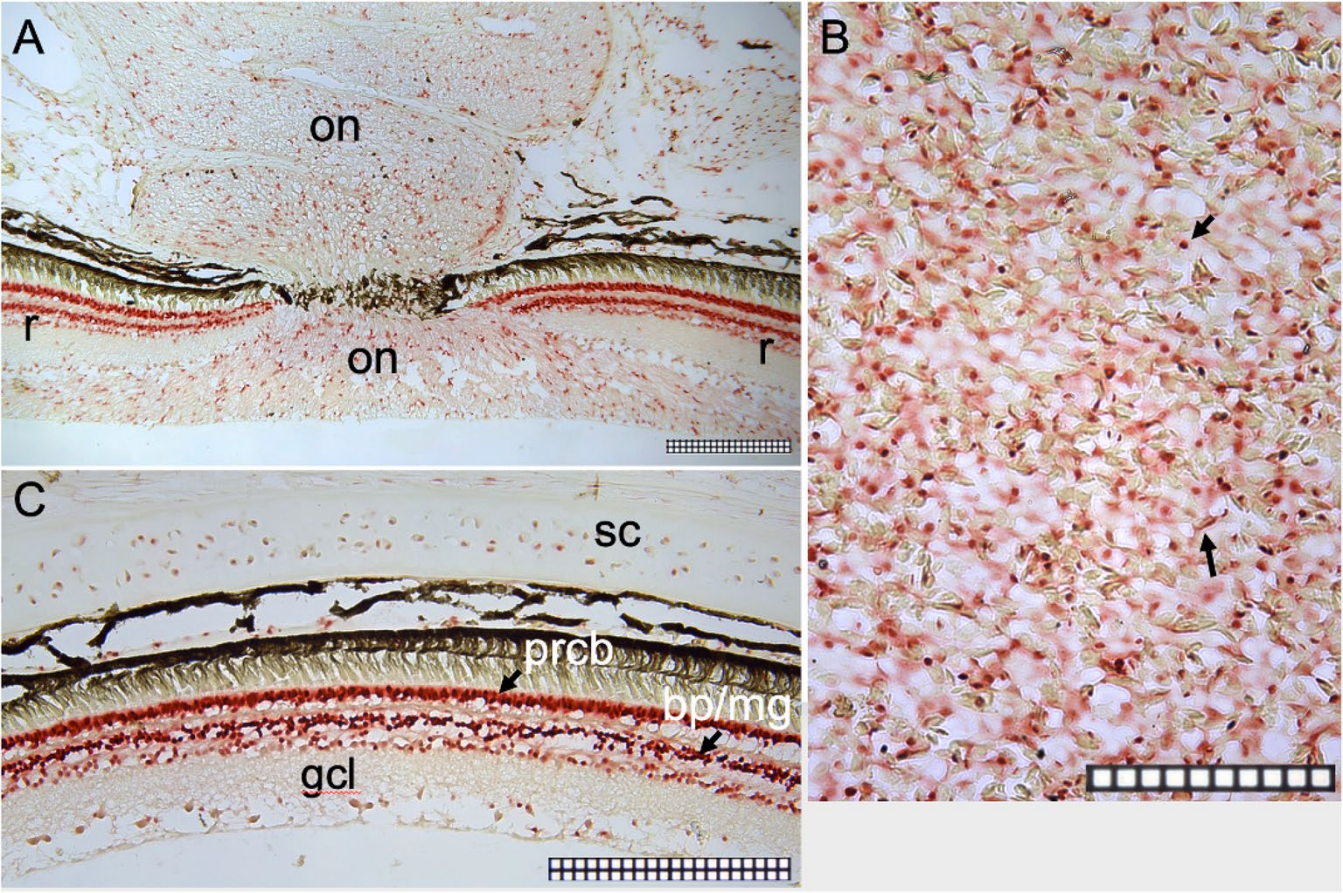
Immunohistochemical analysis of Sox9 expression in normal spotted wolffish ocular tissues. (**A**) and (**C**), Sox9 expression was detected in the nuclei of photoreceptor cell bodies (prcb), in the nuclei of the retinal (r) stratum known to contain both Müller glial cells and bipolar cells (bp/mg), in cells scattered throughout the retinal ganglion cell layer (gcl) and optic nerve tract (on). Sox9 is expressed at very high levels in small leukocytic like cells and flattened endothelial-like cells in the spotted wolffish rete mirabile and choroid body vascular tissues (**B**). (**C**), Sox9 was expressed at detectable levels in chondrocytes within the scleral cartilage (sc). Scale grid subdivisions are 10 µm. Representative images are shown.

Immunohistochemical analyses were performed to test the anti-IgM, anti-PCNA, anti-CD45, and anti-phosphotyrosine antibodies on spotted wolffish head kidney or retinal positive control tissue (Supplemental Fig. 6A-I) while the anti-Sox9 antibody was tested in the spotted wolffish ocular tissues (Fig. 4). We found that Sox9 is expressed in spotted wolffish retina in nuclei of photoreceptor cell bodies and in nuclei present in the retinal stratum known to contain both Müller glial cells and bipolar cells (Fig. 4A,C). Moreover, Sox9 is expressed in cells scattered throughout the retinal ganglion cell layer and optic nerve tract (Fig. 4A). Finally, Sox9 is expressed at very high levels in small leukocytic like cells and flattened endothelial like cells in the spotted wolffish rete mirabile and choroid body vascular tissues (Fig. 4B). Sox9 was expressed at lower but detectable levels in chondrocytes within the scleral cartilage of normal adult spotted wolffish eyes (Fig. 4C).

**Figure 5:**
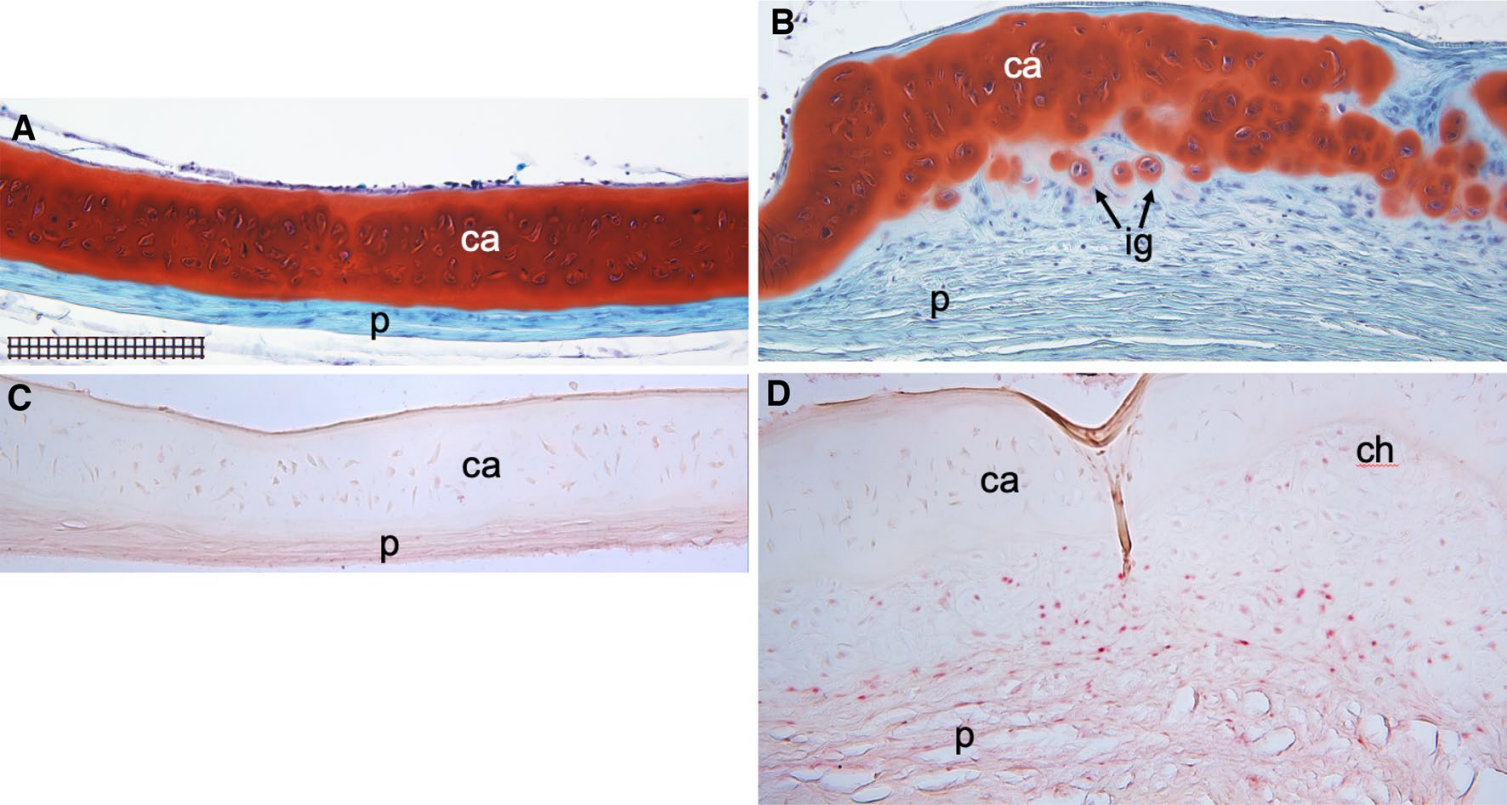
Histochemical and immunohistochemical analysis for PCNA in spotted wolffish scleral skeletal tissues. Representative normal (**A**, **C**) and lesion-like (**B**, **D**) scleral cartilage areas. (**A**) and (**B**), safranin O-fast green staining of areas of sections adjacent to those shown in (**C**) and (**D**), respectively. Safranin O staining (red/orange) marked cartilage matrix. (**B**) Isogenous groups of new chondrocytes (ig, arrowed) starting to secrete islands of cartilage matrix in areas above the perichondrium (p). A higher power X400 view of (**B**) displaying activated maturing chondrocytes is shown in Supplemental Fig. 7. IHCs for PCNA revealed a cellular nuclear staining (red) in perichondrium (p) and the region consistent with the position of the maturing chondrocytes (ch) of the scleral cartilage lesion-like areas of an abnormal wolffish eye (**D**). (**C**) PCNA staining was very low or not detected in other normal regions of the scleral skeleton in the same animal. No counterstain was applied to the sections shown in (**C**, **D**) in order to emphasize the IHC signals. Scale grid subdivisions in (**A**) represent 10 µm, panels (**A**–**D**) magnification: X200. Representative images are shown.

Immunohistochemistry analysis of the spotted wolffish head kidney tissue showed that PCNA expressed with an expected nuclear localization pattern in the parenchyma (Supplemental Fig. 6A). CD45 as detected using the anti-seabass CD45 mAb DLT22^42^, was expressed with an expected cell membranous and cytoplasmic localization pattern in the head kidney parenchyma but not in adjacent skeletal muscle tissue (Supplemental Fig. 6B). Expression of phosphotyrosine was observed in epithelial cell cytoplasms of head kidney renal tubules, in scattered cells in head kidney parenchyma and in endocrine like tissue embedded within the head kidney organ (Supplemental Fig. 6C,D). The anti-lumpfish-IgM IgY antibody we previously used on lumpfish tissues stained spotted wolffish head kidney in a pattern consistent with that observed in lumpfish head kidney^6^ (Supplemental Fig. 6G,H) and strongly stained leukocytic cells in the choroid body/rete mirabile with a pattern similar to Sox9 expression in this tissue (Supplemental Figs. 6J and Fig. 4B). Pre-absorption of the anti-lumpfish IgM antibody with purified lumpfish IgM abrogated the spotted wolffish head kidney IgM staining (Supplemental Fig. 6I). We found no detectable PCNA expression in scleral skeletons of normal healthy spotted wolffish (Fig. 5A,C). Scleral cartilage lesion-like areas contained high levels of perichondrial PCNA expression (Fig. 5B,D). Isogenous groups of chondrocytes were present adjacent to areas with immature chondrocytes displaying pale perinuclear lacunae indicative of cellular activation (Supplemental Fig. 7^29^.

**Figure 6:**
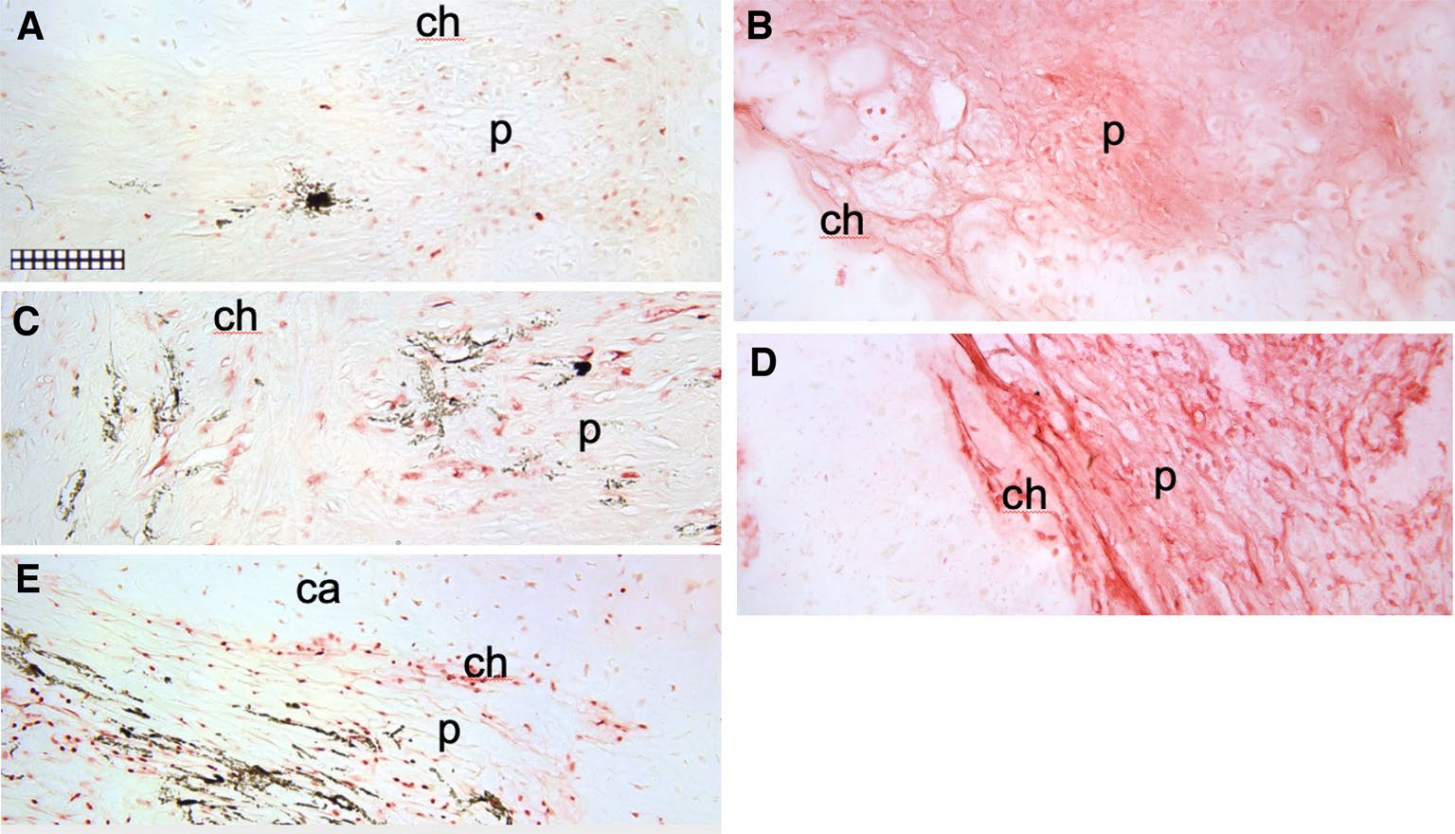
Immunohistochemical analysis of markers of cell proliferation, cell lineage and cellular activation in spotted wolffish scleral skeleton lesion lesion-like tissue. IHCs staining (red) for PCNA (**A**), IgM (**B**), CD45 (**C**), phosphotyrosine (**D**) and Sox9 (**E**) were performed on sections adjacent to the safranin O-fast green stained section shown in Fig. 2I. Areas shown in (**A**), (**C**) and (**E**) encompass the region indicated by the yellow box in Fig. 2I, while areas shown in (**B**) and (**D**) encompass the region indicated by the white box in Fig. 2I. (**A**) Staining for PCNA appeared as a cellular nuclear staining in the perichondrium (p) and some areas consistent with the position of the maturing chondrocytes (ch) (see Supplemental Fig. 8B). (**B**) Staining for IgM appeared in both perichondrium (p) and in regions containing maturing chondrocytes (ch). (**C**) Staining for CD45 appeared in the perichondrium (p) and some areas consistent with the position of the maturing chondrocytes (ch) (see Supplemental Fig. 8A). (**D**) Staining for phosphotyrosine appeared in tracts in the perichondrium (p) projecting into the center of the lesion-like area and in areas consistent with the position of the maturing chondrocytes (ch) (see Supplemental Fig. 10). (**E**) Sox9 appeared as a cellular nuclear staining within the perichondrial (p) tracts projecting into the center of the lesion-like area, in areas consistent with the position of the maturing chondrocytes and in the cartilage (ca) (see supplemental Fig. 9). No counterstain was applied to the sections shown in (**A**–**E**) in order to emphasize the IHC signals. Scale grid subdivisions are 10 µm.

**Figure 7:**
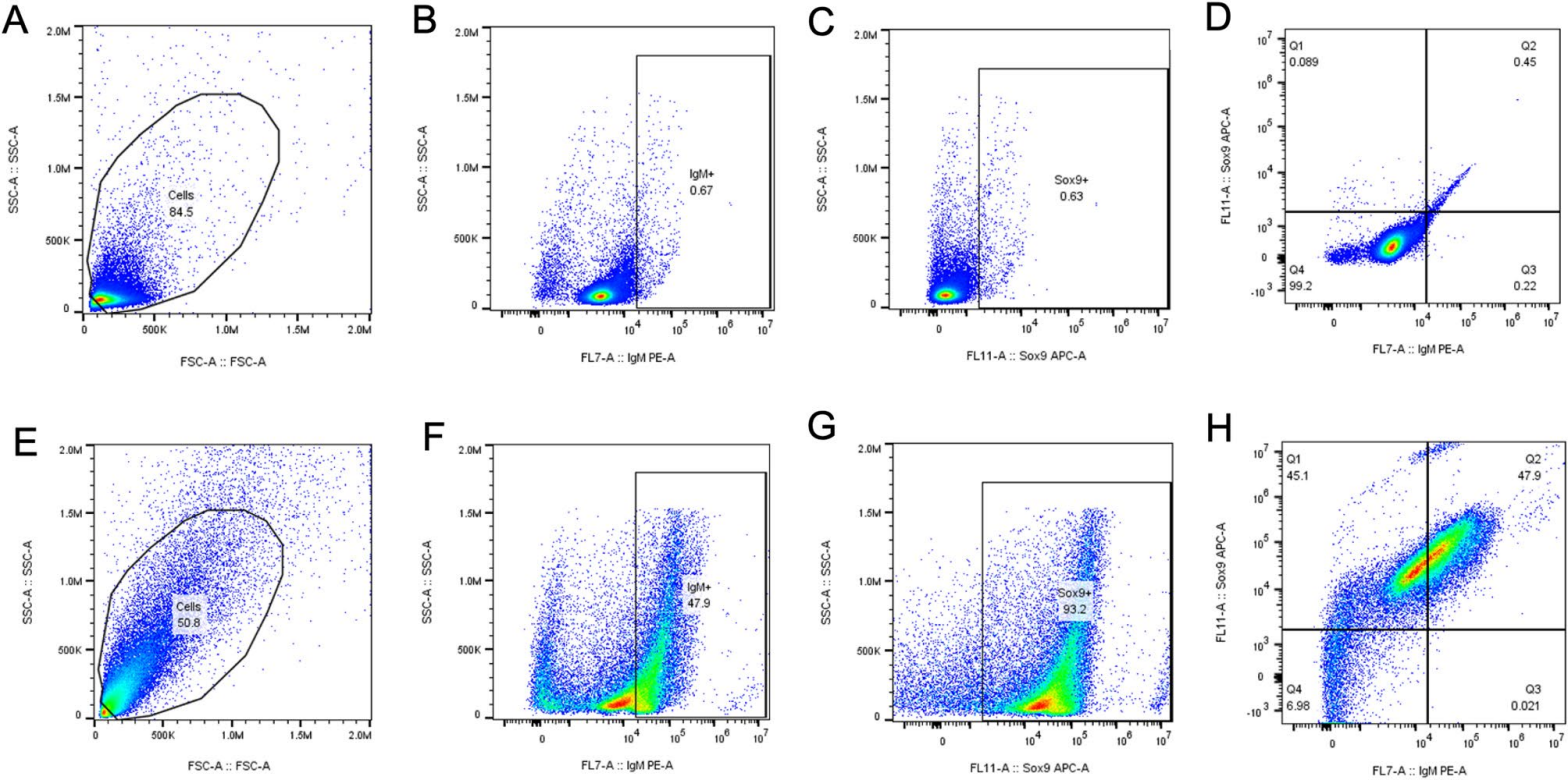
Flow cytometric analysis of IgM and Sox9 in the rete mirabile and choroid body vascular organs of spotted wolffish. Flow cytometric analysis was performed on unstained cell preparations (**A**–**D**) and cell preparations stained with biotin-anti-IgM and rabbit monoclonal anti-Sox9 antibodies using streptavidin-phycoerythrine (PE) and anti-rabbit-alexafluor 647, respectively, as secondary conjugates (**E**–**H**) of the rete mirabile and choroid body vascular organs of spotted wolffish. Stained cells were analyzed for expression of IgM (**F**), Sox9 (**G**) and both IgM and Sox9 (**H**). (**A**), ungated cells. (**F**–**H**), stained cells gaited to exclude cellular debris and analyzed for IgM and Sox9 expression, as indicated. (**B**–**D**), unstained cells gated to exclude cellular debris and gated the same as the stained sets, as indicated. A representative experiment is shown.

We next stained a set of adjacent sections of scleral cartilage lesion-like tissue (a representative section of lesion-like tissue is shown in low power whole globe histological views in Fig. 2H,I) with markers of cell proliferation (PCNA), cell lineage (CD45, IgM for leukocytic lineage; Sox9 for chondrocytic lineage) and an antibody specific for phosprylated tyrosine residues which serves as a marker of cellular activation (Fig. 6). Safranin O staining indicated the near globe-wide scleral cartilage lesion-like tissue traversing the center of the eye from posterior to anterior in the representative specimen shown in Fig. 2H,I. Similarly as shown in Fig. 5, PCNA expression is found mainly in the perichondrial region of the lesion-like tissue (Fig. 6A and Supplemental Fig. 8). CD45 expression appeared in these same areas (Fig. 6C and Supplemental Fig. 8). Both markers stained some cells in the maturing chondrocyte region. The opposite (anterior) side of this lesion-like structure showed similar distribution patterns for PCNA and CD45 (data not shown). Staining for IgM and phosphotyrosine (Fig. 6B,D respectively and Supplemental Fig. 10) also appeared in the perichondrial areas of the lesion-like tissue and was not observed in the maturing cartilage regions. Sox9 expression (Fig. 6E) was detected in the same perichondrial areas of the lesion-like tissue as PCNA, CD45, IgM and phosphotyrosine. However, Sox9 was also detected in some cells in the maturing chondrocyte regions and in tissue peripheral to or outside of the cartilaginous lesion-like tissue (Supplemental Fig. 9). IHC for phosphotyrosine was also performed on sections from normal spotted wolffish eyes (Supplemental Fig. 11). We found that phosphotyrosine is expressed at low but clearly detectable levels in cells in the perichondrium of normal scleral cartilage and in some cases in chondrocytes in and near the edges of segments of scleral cartilage.

**Figure 8:**
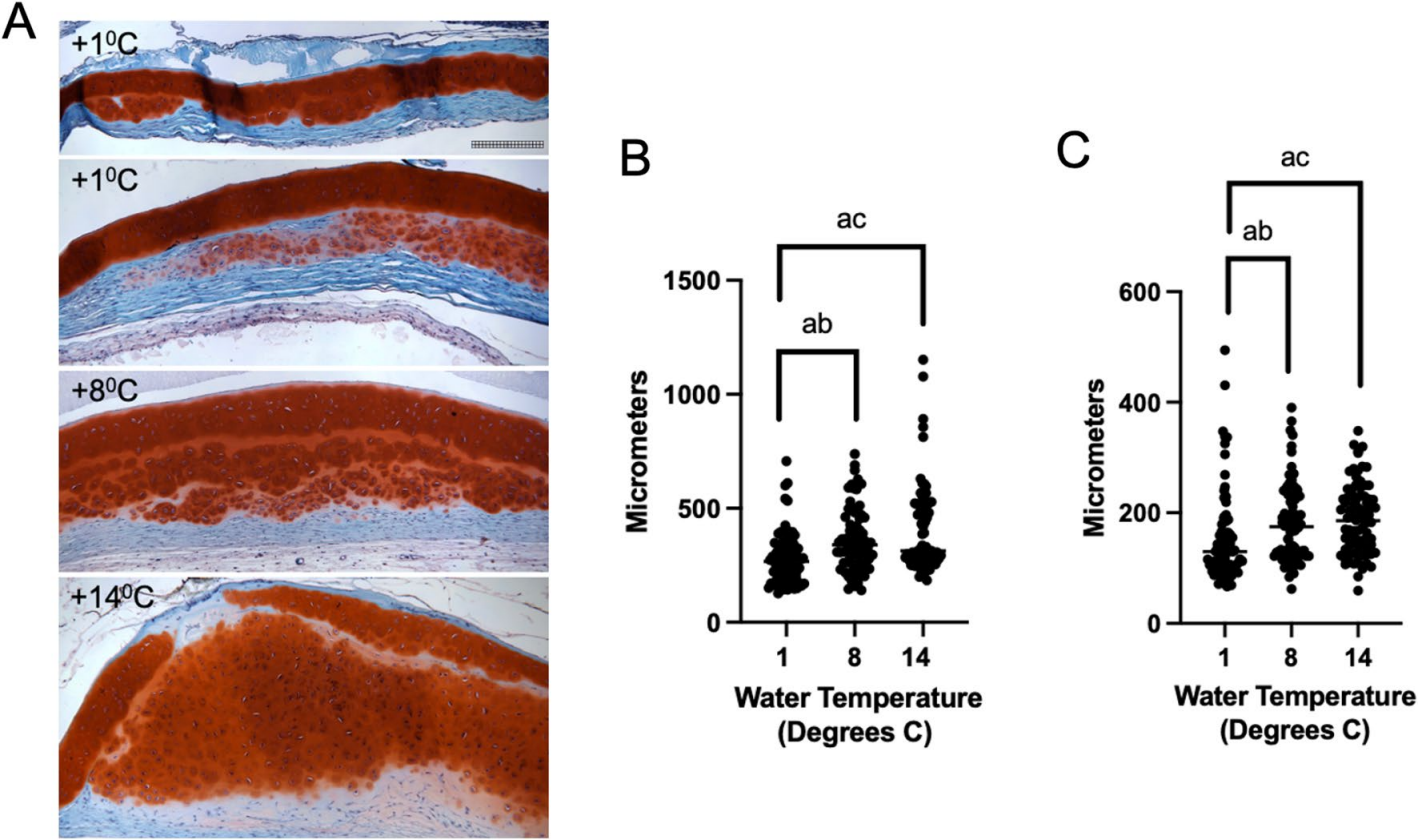
Histochemical morphological analysis of spotted wolffish scleral skeletal tissue after water temperature challenge. (**A**) Safranin O staining of scleral cartilage in spotted wolffish cultured in 1 °C, 8 °C and 14 °C water for 6 weeks as indicated. Two representative cases for 1 °C are shown to display the range of appearance of the tissue. Scale grid subdivisions indicate 10 µm for all panels. Images shown in (**A**) are representative of average values. Measurements of thickness of scleral cartilage including perichondrium (**B**) or not including perichondrium (**C**) for each temperature set (*n* = 4 animals per temperature set). Statistically significant differences (ANOVA, *p* ≤ 0.027) between groups was observed between 1 and 8 °C (ab), and between 1 and 14 °C (ac). No statistically significant differences in scleral cartilage diameter were observed between 8 and 14 °C.

**Figure 9:**
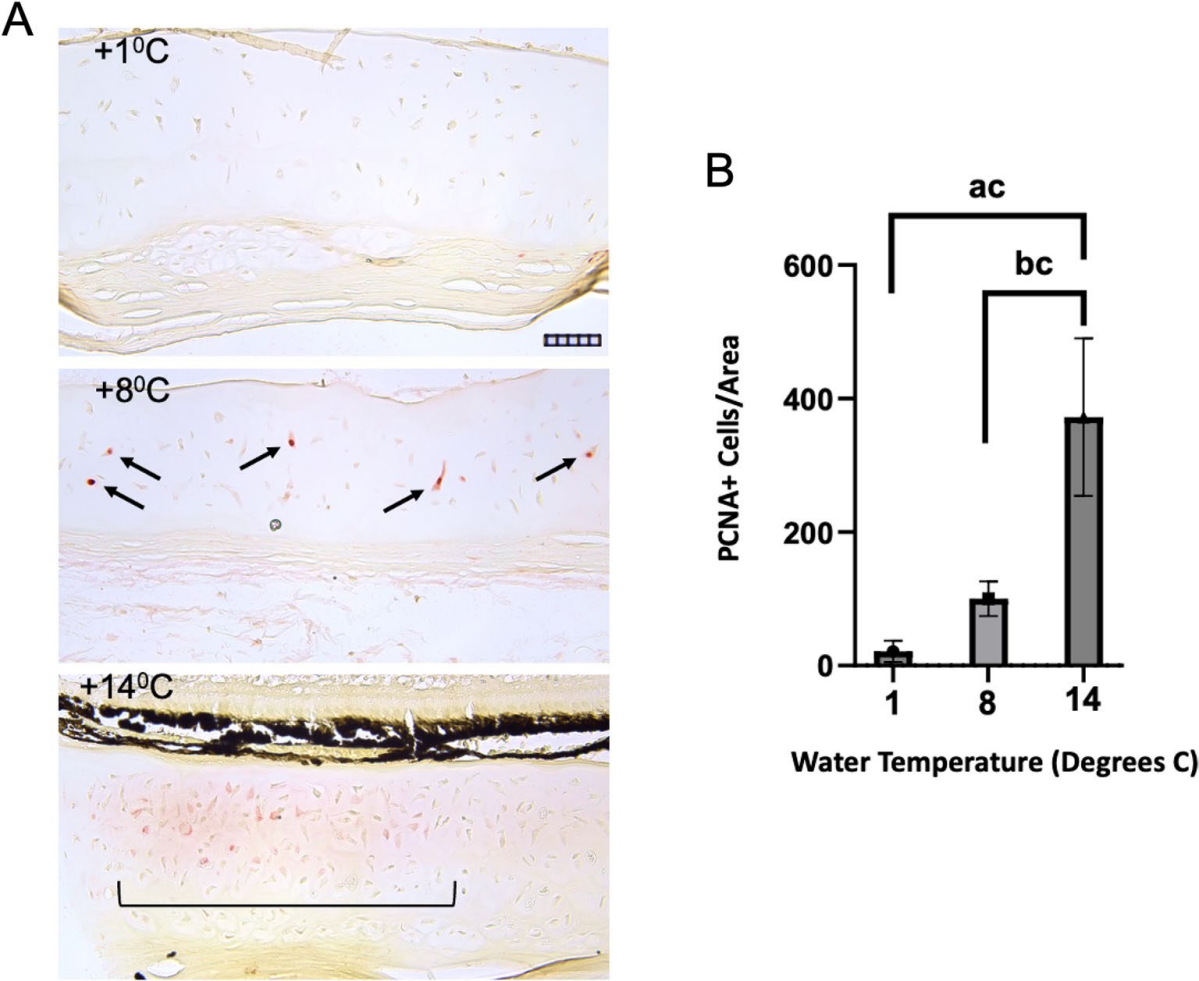
Immunohistochemical analysis of PCNA expression in spotted wolffish scleral cartilage tissue after water temperature challenge. (**A**) Representative PCNA staining of scleral cartilage in spotted wolffish cultured in 1 °C, 8 °C and 14 °C water for 6 weeks as indicated. Arrows in A, 8 °C panel, indicate PCNA positive mature chondrocytes. Bracket in A, 14 °C panel, indicates an area of chondrocytes expressing PCNA. Scale grid subdivisions indicate 10 µm for all panels in (**A**). (**B**) Quantitation of immunohistrochemistry analysis of PCNA positive cell number per scleral cartilage area (*n* = 4 animals per temperature set). Values are expressed as a percentage relatively to the average 8 °C value. Statistically significant difference (ANOVA, *p* ≤ 0.0044) was observed between 1 and 14 °C (ac), as well as between 8 and 14 °C (bc).

Our IHC analyses were single marker stainings. In order to further explore the expression of immune lineage and chondrocytic cell markers on spotted wolffish ocular cell populations, we performed flow cytometry analysis on cells prepared from choroid body and rete mirabile tissues from normal spotted wolffish without ocular abnormalities. These tissues were chosen with the rationale that they represent an internal reservoir of immune lineage cells in the teleost eye. Flow cytometric analysis was performed using spotted wolffish rete mirabile and choroid body vascular organ cell preparations. Unstained cell preparations (Fig. 7A–D) and cell preparations stained with biotin-anti-IgM and rabbit monoclonal anti-Sox9 antibodies using streptavidin-phycoerythrine (PE) and anti-rabbit-alexafuor 647, respectively, as secondary conjugates, are shown (Fig. 7E–H). Flow cytometric analysis of the rete mirabile and choroid body vascular organs of normal spotted wolffish eyes revealed that approximately 93% of the examined cells exhibited Sox9 expression (Fig. 7G), while approximately 48% displayed IgM expression (Fig. 7F). Among the Sox9 expressing cells, approximately 48% concurrently coexpressed IgM (Fig. 7H,Q2).

### Effects of temperature on chondrogenesis of spotted wolffish eyes

Since it is not possible to define a common factor causing the development of the scleral cartilage lesion-like tissue we identified in wolffish presenting with various ocular abnormalities, we tested the effects of increased water temperature as a controllable stress or insult to spotted wolffish eyes with the rationale that a range of stresses and insults might trigger abnormal scleral cartilage growth in this animal. The temperature range was chosen to approximate temperatures in the wild (2 to 8 °C)^10^, but also slightly exceeding temperatures encountered in the culture of spotted wolffish which it is normally 4 to 12 °C^14^. We observed a direct correlation between water temperature and the level of scleral cartilage chondrogenesis as measured by safranin O staining in cohorts of spotted wolffish exposed to 1 °C, 8 °C and 14 °C water temperatures for 6 weeks (Fig. 8). As we observed in other specimens of normal adult spotted wolffish (Figs. 1, 2), low levels of scleral chondrogenesis were occasionally observed at the temperature of 1 °C (see 2 separate examples from the 1 °C cohort in Fig. 8A). Exposure to higher water temperatures of 8 °C and 14 °C led to a significant increase in scleral cartilage chondrogenesis relative to 1 °C as measured by safranin O staining of scleral cartilage tissue area (Fig. 8B,C) and analysis of variance (ANOVA, *p* ≤ 0.027). Significant increases of scleral cartilage tissue thickness as a function of increased temperature were measured either including or not including the perichondrium. We also assessed the expression of PCNA protein in these sets of tissues. Animals held at 1 °C rarely showed detectable PCNA staining in scleral cartilage. Exposure to increasing water temperature of 14 °C led to a significant increase (ANOVA, *p* ≤ 0.0044) in the numbers of cells expressing PCNA in the scleral cartilage tissue area (Fig. 9), while the total cell density was not changed (not shown).

## Discussion

To begin to address the objective of providing knowledge on the eyes of spotted wolffish for which a scientific literature is lacking, we focused herein on scleral chondrogenesis in normal control and ocular insult paradigms in cultured spotted wolffish. Our results provide new knowledge on spatial tissue expression patterns of chondrogenesis markers during scleral chondrogenesis responses in this animal. Our results indicate that spotted wolffish possess an inherent scleral chondrogenesis response that may be sensitive to temperature. This work advances the fundamental knowledge of teleost ocular skeletal systems. Moreover, since there is, to our knowledge, no literature on the morphology and molecular expression characteristics of spotted wolffish eyes, our work advances the knowledge base on spotted wolffish in comparison with existing literature. Our work has some limitations. We cannot legally access wild specimens of this animal for comparative studies. We face the contraint of a single aquacultured colony as a study population. Our work nonetheless provides a basis to further explore the visual system of spotted wolffish, which are designated a threatened species in Canada. In previous work, we characterized the visual system of the North Atlantic lumpfish^1–7^. The work herein on spotted wolffish suggests some differences (scleral cartilage thickness, shape and reactivity) and similarities (expression of cellular and immune markers PCNA, IgM and CD45), between the two species.

The ocular histopathologies we observed in spotted wolffish were low in frequency and may be related to in-tank ocular trauma. However, some animals with earlier stages of pathology could have escaped notice in routine screening. Alternatively, the ocular histopathologies we observed in spotted wolffish could be due to genetically based ocular abnormalities. Since the spotted wolffish genome remains uncharacterized, genomic work must be completed before any hypotheses or conclusions can be made in this regard. Myopia in humans is an example of how ocular shape changes can directiy affect light ray refraction and thus visual acuity. Scleral cartilage pathology in spotted wolffish could conceivably alter the visual function capacity of the spotted wolffish eye if the pathological changes and deformation impact the physical shape of the sclera, closely underlying reflective membranes such as tapetum lucidum, the function of retinal tissue and/or the integrity of other anterior tissues important for ocular function. We speculate that such potential changes, if severe enough, could affect visual acuity which could compromise the animal’s abilities in both wild and cultured settings and thus could be relevant to industrial aquaculture operations. Since the animals we assessed for tank-derived ocular insult were on average smaller than those we used to initiate the temperature challenge studies, it remains possible that ocular abnormailities impacted visual acuity in these animals. However, all cases of ocular abnormalities were unilateral and tankmates of similar average weight not showing ocular abnormalities were used as controls. Bilateral ocular abnormalites would likely be more impactful to the health of the animal.

Despite the lack of genomic resources for spotted wolffish, the panel of anti-teleost and/or anti-mammalian monoclonal antibody reagents we used to obtain the results herein was validated on spotted wolffish eye and head kidney tissues, the latter of which is a primary lymphoid organ in teleost^43,44^. Pre-absorption of the anti-lumpfish IgM antibody with purified lumpfish IgM abrogated the spotted wolffish head kidney IgM staining, further demonstrating cross reactivity. Spotted wolffish IgM can also be purified from spotted wolffish blood, the signal can be detected by Western blotting with the anti-lumpfish IgM antibody and the signal is abrogated by pre-absorption with purified spotted wolffish or lumpfish IgM. Geimsa staining of adjacent sections confirms the cytoplasmic localization of the phosphotyrosine staining in the head kidney parenchyma and renal tubule epithelium and this is consistent with previously reported cytoplasmic phosphotyrosine staining in epithelium^41^. The endothelial staining for phosphotyrosine we observed in wolffish tissues is consistent with previous findings in mammal^41^. All IHC stains were also performed with the primary antibody omitted as negative control (see Supplemental Fig. 5) and always showed no staining. These controls also ruled out the possibility of the antibodies binding nonspecifically to macrophage cells via Fc receptors since all second antibody reagents were also whole immunoglobulins. Western blotting for spotted wolffish PCNA, CD45, Sox9 and Tubulin further validated the utility of the panel of anti-teleost and/or anti-mammalian monoclonal antibody reagents we used to obtain the results on spotted wolffish tissues herein. Although there were differences in the molecular weights of spotted wolffish versus lumpfish anti-CD45 immunoreactive bands on western blotting, these differences could be due to either species specific differential splicing or posttranslational modifications as we have described previously for sea bass versus lumpfish CD45 and as others have described in other species^4,45^. The Basic Local Alignment Search Tool at National Center for Biotechnology Information (BLAST) indicated 94.8% and 90.7% protein sequence identities for seabass CD45 and Sox9 compared to the lumpfish and sablefish (*Anoploploma fibria*) genomes, respectively, which were used as a proxies for spotted wolffish in the absence of spotted wolfish genomic information. We described previously that the CD45 phosphatase domains are very conserved between taxa. Although the R0 and ABC domains are not typically conserved between species and are in different fish clusters between lumpfish and seabass, there are still similarities in this domain between lumpfish and seabass which supports antibody cross reaction with spotted wolffish^4^. Finally, our IHC validation experiments clearly show DLT22 antibody anti-CD45 staining in spotted wolffish head kidney tissue where one would expect such staining in the significant immune lineage parenchymal cell component in this primary lymphoid organ but not in spotted wolffish muscle tissue where one would not expect any appreciable immune lineage cell component in the parenchymal muscle tissue (Supplemental Fig. 6B).

The Sox9 expression we observed in normal spotted wolffish retina, ganglion cell layer, optic nerve tract, posterior ocular vascular tissues and scleral cartilage suggest that populations of different lineages of cells expressing Sox9 reside in the normal spotted wolffish eye. The Sox9 protein is a transcription factor that plays many roles throughout vertebrate development, and has been identified as being pivotal in neural crest formation, required for human male sex determination, and essential for mesenchymal condensation prior to chondrogenesis by regulating the expression of downstream target genes^46^. Sox9 expression levels have been shown to positively correlate with chondrogenesis potential in studies in mammals^47^. A zebrafish ortholog of Sox9 has been described to be essential for cartilage development^48^. Loss of function of sox9a results in considerable cartilage defects in zebrafish, whereas loss of function of sox9b did not result in the same defects^49^. Due to genome duplication events in the teleost lineage^50^, teleost fish are thought to have two Sox9 paralogs, Sox9a and Sox9b, which are regulated by different pathways and are suggested to have a combined expression pattern that corresponds to the ancestral Sox9 function^51^. Our Western blotting findings suggest that there is one Sox9 protein in spotted wolffish ocular tissues and that this protein cross reacts with mammalian Sox9. Our results are consistent with what has previously been considered a pleiotropic role for Sox9^51–55^ but also provide new evidence on the possibility that Sox9 might be more widely utilized in the spotted wolffish eye. Much of the literature regarding the complexities of Sox9 expression during chondrogenesis examine in vitro temporal expression, often in mesenchymal stem cells^56–59^. Our results provide knowledge of differing Sox9 spatial tissue expression patterns during chondrogenesis in normal control and ocular insult paradigms. A better understanding of the tissue spatial regulation of Sox9 during in vivo chondrogenesis in health and disease will help elucidate the complex role of Sox9 in chondrogenesis.

We observed a significant number of Sox9 + cells scattered throughout the scleral cartilage lesion-like areas in abnormal spotted wolffish eyes and in areas in which PCNA, CD45, IgM and phosphotyrosine are also highly expressed. The perichondrial PCNA expression in these lesion-like areas of tissue indicates perichondrial proliferation, the pale perinuclear lacunae in newly formed chondrocytes indicates chondrocytic activation and the classical grouping into isogenous groups of chondrocytes surrounded by islands of safranin O fast green-positive cartilage matrix indicates active de novo cartilage formation^29^. It is conceivable that the Sox9 + cells in the spotted wolffish scleral cartilage lesion-like tissue are chondrocyte progenitors that, if present in a permissive microenvironment, might undergo precartilagenous condensation and chondrogenesis^59^. Our results demonstrate Sox9 expression in the retinal layer containing Müller glial cells. Since Müller glial cells are leukocytic lineage cells that mediate a range of homeostatic as well as inflammatory processes in the eye, it is conceivable that the origin of the Sox9 + cells scattered in the lesion-like areas in these spotted wolffish eyes might be Müller glia that have relocated to sites of pathology in these eyes.

Our results suggest that the chondrogenesis occurring in spotted wolffish scleral cartilage tissue might be supported by CD45 + cells as well as IgM since Sox9 + , PCNA + perichondrial regions underlying newly generated cartilage contained CD45 + cells and IgM. CD45 has been shown to be expressed by a significant proportion of human induced chondroprogenitor (hiCPC) cell lines^60^. Moreover, previous studies have shown that expression of chondrogenic genes *col2a1* and *sox9* is enhanced during chondrogenesis occurring in the presence of hematopoietic-lineage-derived CD45 + cells^60^. These studies support a hypothesis that CD45 + cells might, in specific cases, support or drive chondrogenic cellular potential. Using flow cytometry of tissue harvested from the rete mirabile/choroid body of spotted wolffish, we observed separate populations of IgM + cells, Sox9 + cells and cells concurrently expressing IgM and Sox9. These results further support a pleiotropic role for Sox9 in hematopoietic cells in spotted wolffish ocular tissues. B lymphocytes are known to play a role in rheumatoid arthritis^61^. Sequestration of IgM inside cartilage tissue has been described as a feature in patients with rheumatoid arthritis and has been hypothesized to be associated with cartilage degeneration in this disease process^62^. Chondrocytes have been reported to bind IgM^63^. Immune complexes containing IgM from rheumatoid arthritic serum and synovial fluid has been hypothesized to modulate the growth characteristics of chondrocytes^64^. These studies offer evidence that chondrogenesis might be driven by B lymphocytes, IgM or both. IgM + marine teleost B lymphocytes are phagocytic and it is thought that this teleost cell lineage could serve the phagocytosis function of the evolutionarily diverged macrophage^65^. Phagocytic B cells in teleosts are capable of presenting antigen fragments to CD4 + cells, initiating the adaptive immune response and bridging the gap between innate and adaptive immunity^66^. In humans with osteoarthritis, B cells appear to both impair and enhance cartilage repair through promotion or inhibition of inflammation^67^. Mammalian B1a and B1b lymphocytes have the ability to both phagocytose large particles and undergo antigen presentation^68,69^. The presence of IgM + cells in spotted wolffish scleral cartilage lesion-like tissue is intriguing and may offer new insight to the role of the immune system and phagocytosis in particular, in response to ocular insults. Among the Sox9 expressing cells in the spotted wolffish rete mirabile/choroid body tissue, we found that approximately 48% concurrently coexpress IgM. It is thus conceivable that a dormant chondrogenesis pathway in normal spotted wolffish eye might be mediated by this proportion of ocular immune /chondrogenic like cells.

In temperature challenge experiments, performed on larger animals, low but observable levels of scleral chondrogenesis were observed in some fish housed at the temperatures of 1 °C as shown in Fig. 8A. This level of scleral chondrogenesis is similar to what we observed in some normal juvenile and early adult spotted wolffish housed at 6–10 °C. The significant increase in scleral cartilage chondrogenesis as measured by safranin O staining in adult spotted wolffish exposed to water temperatures of 14 °C was similar to the levels of scleral cartilage chondrogenesis we observed in some of the cases using the ocular abnormality paradigm. The average weight of the fish from the 14 °C set was not significantly higher than that from the lower temperature sets (Supplemental Fig. 2). Therefore, overall increased fish growth does not seem to be the cause of the increased scleral cartilage tissue mass. Proliferating cell nuclear antigen is a protein which partners with DNA polymerase, is expressed in G1, S and early G2 phases of the cell cycle and specifically marks proliferating cells in ocular and other tissues (37, 40). The significant increases in numbers of PCNA positive cells in both prechondrocytes and mature chondrocytes in the scleral cartilage from the increased temperature cohorts indicates that the cells present in these tissues acquire a proliferative phenotype with increasing temperature. This argues against the increase in scleral cartilage thicknesses being the result of acellular material expansion or deposition alone as causing the thickening. Taken together with the increased scleral cartilage thickness (Fig. 8), the increase in numbers of PCNA + cells but not total cell density with increasing water temperatures supports the conclusion that a process of chongrogenesis is occurring in these tissues, with expansion of a chondrocytic cell population in combination with deposition of new cartilage matrix. Our overall findings for PCNA expression in both the ocular abnormality and temperature induction paradigms indicate that groups of spotted wolffish of different average weight ranges display an inherent potential for scleral chondrogenesis which is associated with chondrocyte proliferation. In the wild, spotted wolffish normally inhabit waters with temperatures that could be lower than 5 °C but could also span ranges from this up to 10 °C^10,11^. Regardless of the range of temperatures spotted wolffish encounter in the wild versus conditions optimal for an industrial cultured fishery, our results provide evidence that important ocular structures might be sensitive to higher temperatures. While rising ocean temperatures would probably be represented by lower temperature difference ranges, the potential impact of temperature changes on the integrity of ocular health in wild spotted wolffish might be worthy of further, more detailed study.

Both chondrogenesis and Sox9 expression have been found previously to be affected by temperature^69–71^. The mechanisms by which homeotherm limb growth is directly proportional to environmental temperature is not well understood^69^. However, for ectotherms such as teleost fish, elevated temperature is often associated with higher rates of growth, until an eventual plateau is reached^72,73^. Growth and rearing at sustained elevated temperatures has been implicated in causing vertebral deformities^74,75^. Juvenile spotted wolffish display higher growth rates when reared at 6 °C compared to 12 °C, and optimal growth temperature decreases with increasing size and age^76^. Spotted wolffish have been classified as stenothermal, thus possibly tolerating only small ranges of temperatures^76^. When raised at 13 °C, Atlantic wolffish display skeletal abnormalities^75^ but we are not aware of any published data on spotted wolffish. The purpose, if any, of the scleral cartilage response to increased water temperature is not known but might be indicative of a tissue specific stenothermal nature of spotted wolffish, which deserves further study. This work could be relevant to the health of spotted wolffish and could possibly help guide conservation efforts in the wild in the context of ocean health, climate change and increased ocean water temperatures. The work could also be relevant to industrial aquaculture applications where spotted wolffish might be held at a range of temperatures.

## Methods

### Spotted wolffish tissue

#### Spotted wolffish source, housing and animal numbers

All studies were performed in accordance with the guidelines of the Canadian Council on Animal Care and protocols were approved by Memorial University of Newfoundland’s Institutional Animal Care Committee (protocol #17-03-RG; #18-01-JS; #18-02-JS). While much of the data and results presented herein are qualitative in nature, this study was otherwise performed and reported in accordance with ARRIVE guidelines.

Spotted wolffish of approximately 2 years of age were obtained from cultured stocks at the Dr. Joe Brown Aquatic Research Building (JBARB), Department of Ocean Sciences (DOS), Memorial University. The stocks were grown under experimental holding conditions from fish that came originally to JBARB as juveniles from Amar Seafoods Ltd. The animals had been resident of JBARB for 14 months prior to February 2023. Juvenile fish sampled had been housed in 6–10 ºC in 95–100% air saturated filtered and ultraviolet treated sea water and were in the weight range of 100–200 g. All spotted wolffish described in this manuscript were of mixed or unknown sex. We designated spotted wolffish with no gross or eye abnormalities as “normal”. Animals displaying grossly observable eye abnormalities were harvested for microscopic analysis and tank mates without ocular abnormality were harvested as controls. The average weight of the spotted wolffish we had access to for tissue harvesting for ocular analyses was 307 g. We assessed paraffin embedded fixed material from 3 normal spotted wolffish (i.e., spotted wolffish with normal grossly observable ocular features) and 4 spotted wolffish harboring a range of grossly observable ocular abnormalities. Four additional normal juvenile spotted wolffish were used for flow cytometry and western blotting experiments. Abnormal tissue observed microscopically in the scleral cartilage was designated “scleral cartilage lesion-like tissue”.

Separate groups of adult spotted wolffish (average weight of 780 g at harvest) were randomly sampled from tanks of fish that had been stocked at 25 kg / m2 of tank bottom in which there were 100 fish per tank and which had been acclimated to 1 °C, 8 °C and 14 °C for a period of 6 weeks (see Supplemental Figs. 1–3). These fish were sampled from a population that was being exposed to these temperatures in a separate study designed to assess metabolic parameters in relation to the ability of this species to tolerate warmer temperatures. Four animals per temperature were used in histochemical and PCNA quantitative morphometric and immunohistochemical analyses. The temperature range was chosen to approximate the temperatures in the wild (2 to 8 °C)^10^, but also slightly exceeding temperatures encountered in the culture of spotted wolffish which it is normally 4 to 12 °C^14^. For the fish used in the temperature experiment, these fish were housed in a separate building (the “Back Tank Building”) with a photoperiod of 16 h light: 8 h dark, sea water water air saturation levels of ~ 100–115%. Flow into the tanks was at 20 L / min. Only one tank (2.0 m in diameter, filled to 40 cm in depth) was used per temperature. Temperature control was + /−0.2 oC (see Supplemental Figs. 1–3). Since the temperature challenge experiments were performed over an extended period of 6 weeks (Supplemental Figs. 1–3), the animals in these cohorts were monitored carefully on a daily basis by DOS staff for any signs of health problems.

Eye and head kidney tissues from adult lumpfish were used for controls and sourced from DOS, Memorial University of Newfoundland, Canada or from archived material in our laboratories. The lumpfish were kept according to established conditions^77^, at 10 °C in 500 L tanks supplied with 95–100% air saturated and ultraviolet treated filtered flow-through seawater and an ambient photoperiod.

#### Tissue processing

Fish were euthanized using a lethal dose of TMS/ MS-222 at 400 mg/l. Eyes were inspected prior to enucleation for abnormalities, carefully excised, fixed in 4% paraformaldehyde for 72 h and embedded in paraffin blocks. Alternatively, ocular tissues were dissected and snap frozen for protein isolation.

### Histology, immunohistochemistry (IHC) and microscopy

Paraffin blocks of spotted wolffish eye tissues were sectioned onto positively charged slides. Slides were processed for immunohistochemistry and / or stained with standard hematoxylin and eosin (H&E) or safranin O/ fast green. A procedure published by University of Rochester Medical Center’s Center for Muscoluskeletal Research was followed for the safranin O/ fast green staining^78^.

Immunohistochemistry for cell markers was validated by both IHC and western blot (Supplemental Figs. 5, 6; Fig. 4). PCNA IHC was performed using mouse monoclonal antibody (mAb) PC10 (Santa Cruz Biotechnology Inc.) at a 1:50 dilution. Phosphotyrosine IHC was performed using mouse monoclonal antibody 4G10 (Cell Signaling Technology Inc) at a dilution of 1/300. CD45 IHC was performed as we described previously^4^ using mouse monoclonal antibody DLT22 which is raised against sea bass (*Dicentrarchus labrax*) CD45^42,79^. IgM IHC was performed using a custom anti-lumpfish IgM antibody produced in chicken in collaboration with Somru BioScience (Charlottetown, PEI, Canada) as previously described^4,6^.

We also performed IHC for Sox9^80^. Sox9 IHC was performed using rabbit monoclonal antibody D8G8H (Cell Signaling Technology) according to previously published methodology at a dilution of 1:50^81^.

The IHC procedure for PCNA was as we described previously^1^ with the addition of an antigen retrieval step. Antigen retrieval was also used for the phosphotyrosine and IgM IHC. After deparaffinization, sections were processed for the antigen retrieval step using a Tris- Ethylenediaminetetraacetic acid (EDTA) buffer (10 mM Tris base, 1 mM EDTA solution, 0.05% Tween 20, pH 9.0) at 95 °C for 5 min. The IHC procedure for Sox9 involved an antigen retrieval step using a 10 mM sodium citrate buffer with 0.05% Tween 20, pH 6.0, for 40 min at 97 °C.

For morphometrics of scleral cartilage tissue, measurements of thickness of scleral cartilage (including or excluding the perichondrium) were made from 4 animals for each temperature set. Sixteen images from each temperature were uploaded into ImageJ Fiji and the thickness was manually measured. Five measurements from each image, for a total of 80 measurements per temperature, were used for one way ANOVA to determine statistical significance using GraphPad Prism Version 9. For quantitative morphometrics of scleral cartilage PCNA positive cell number per area, images were uploaded into ImageJ Fiji where the area was manually outlined for calculation and the number of stained cells was manually counted. Four to 5 high power fields per specimen were counted in 4 separate quantitation experiments for all 3 temperatures in 4 separate animals. The mean number of PCNA stained cells per unit area in scleral cartilage of spotted wolffish cultured at different temperatures were expressed as a percentage normalized to that of the mean of a reference 8 °C specimen and as a percentage of the average 8 °C group.

### Spotted wolffish IgM purification and cross-reaction verification

#### Wolffish IgM isolation

Wolffish IgM was purified from serum using an immobilized mannan binding protein (MBP) column kit (ThermoScientific, USA) according to manufacturer’s protocol, with the following modifications^82^. The elution buffer was incubated overnight at 4 °C before elution. All IgM fractions were pooled and dialyzed with 20 mM Tris–HCl (pH 8.0) via dialysis cassette (10,000 Da MWCO, ThermoScientific, USA). The purity of the IgM was evaluated by sodium dodecyl sulfate—polyacrylamide gel electrophoresis (SDS-PAGE) and Coomassie blue staining.

#### Western blotting

The chicken IgY anti-lumpfish-IgM antibody was used at a dilution of 1:10,000 to verify the identity of the purified IgM by Western blotting. A rabbit anti-chicken-IgY-AP antibody (EMD Millipore Corp, USA) was used as secondary antibodies at 1:5,000 dilution. The Western blot signals were developed with a BCIP/NBP solution (VWR Lifescience, USA). To verify the specificity of the anti-IgM Western blot signal, isolated wolffish IgM and chicken anti-lumpfish-IgM antibody were preincubated at 4 °C overnight. This IgM-antibody mixture was then used in place of the primary antibody for Western blotting.

For the other Western blottings, a positive control U-2 OS human osteosarcoma epithelial cell line, acquired from American Type Culture Collection (Manassas, Virginia), was used. Tissue and cell line protein lysates were prepared and protein amounts quantified for Western blot analyses using previously described procedures^4,83, 84^.

Anti-seabass (*Dicentrarchus labrax*) CD45 (mAb DLT22), which we have used previously for western blotting in lumpfish^4^, was used at 1:20 dilution, Sox9 antibody was used at 3 ug/ml, while PCNA antibody PC10 was used at 0.13 μg/ml. Tubulin mouse monoclonal DM1A (Sigma-Aldrich, St. Louis, Missouri) was used at 1 μg/ml to monitor for protein integrity and as loading control. A LumiGLO Reserve Chemiluminescent Substrate Kit (Mandel Scientific, Guelph, Ontario) or a Clarity Western ECL Substrate (BioRad, Hercules, California) were used to develop the Western blot analyses.

Based on the predicted molecular weights of teleost CD45, Sox9, PCNA and Tubulin, the western blot membrane used for Fig. 3 was divided into four portions each containing the molecular weight range of the four proteins (CD45, Sox9, PCNA and Tubulin) being probed. Antibody incubations were performed separately for the four proteins (CD45, Sox9, PCNA and Tubulin) on these portions of filter membrane. Original unprocessed filter membranes used to produce the cropped images shown in Fig. 3 are shown in Supplemental Fig. 4.

### Spotted wolffish ocular tissue flow cytometry

Pairs of eyes from individual fish were carefully enucleated and cut into posterior/anterior halves. The posterior was placed into a 35 mm petri dish into 2 ml of K-EDTA-phosphate buffer saline (PBS) where rete mirabile and choroid body tissues were isolated from retina and scleral tissues. The rete mirabile and choroid body tissues were gently dispersed into a single cell suspension. The tissue was next passed through a cell strainer (35 μm pore size). The cell suspension volume was measured and divided into 3 equal portions to be incubated on ice for 30–45 min either without antibody (unstained control and avidin-phycoerythrin [PE] control) or with biotinylated chicken anti-lumpfish IgM antibody (anti-IgM). Cells were washed, pelleted and resuspended with 1 ml K-EDTA-PBS. Avidin-PE was then added to each of the anti-IgM and avidin-PE control tubes and incubated on ice in the dark for 30 min. After another wash, pellets were resuspended in 250 μL K-EDTA-PBS and 250 μl 4% PFA. IgM flow cytometry was conducted on 100,000 cells. For Sox9 double staining, 0.1% Triton X-100 (v/v) was added to the anti-IgM samples to permeabilize the cells and a 1:100 dilution of rabbit monoclonal anti-Sox9 antibody added. Samples were then incubated at 4 °C overnight in the dark. A donkey anti-rabbit IgG conjugated to Alexa Fluor 647 (Fisher Scientific, Ottawa, ON) was added to the anti-IgM/anti-Sox9 samples. Samples were incubated at 4 °C for 2 h in the dark. After washing, pellets were resuspended in 250 μl K-EDTA-PBS. IgM/Sox9 flow cytometry was conducted on 100,000 cells on a Beckman Coulter Cytoflex flow cytometer.

### Ethical approval

All studies were performed in accordance with the guidelines of the Canadian Council on Animal Care and protocols were approved by Memorial University of Newfoundland’s Institutional Animal Care Committee (protocol #17-03-RG; #18-01-JS; #18-02-JS).

### Supplementary Information

Supplementary Information.

## Data Availability

Much of the data presented herein is qualitative anatomical, histological or immunohistochemical data. The supplemental materials file contains several examples of broader regions of the stained tissue sections used to produce the figures. Otherwise, the datasets used and/or analysed during the current study are available from the corresponding author on reasonable request.
