# Supplementary material for: Increased water temperature contributes to a chondrogenesis response in the eyes of spotted wolffish
Source: Sci Rep. 2024 May 31;14:12508. doi: 10.1038/s41598-024-63370-8 (PMC11143355; doi:10.1038/s41598-024-63370-8)
Supplement: Supplementary file 1 — Supplementary Information. [file 41598_2024_63370_MOESM1_ESM.pdf]

Supplemental Figures and Legends

Supplemental Figure 1

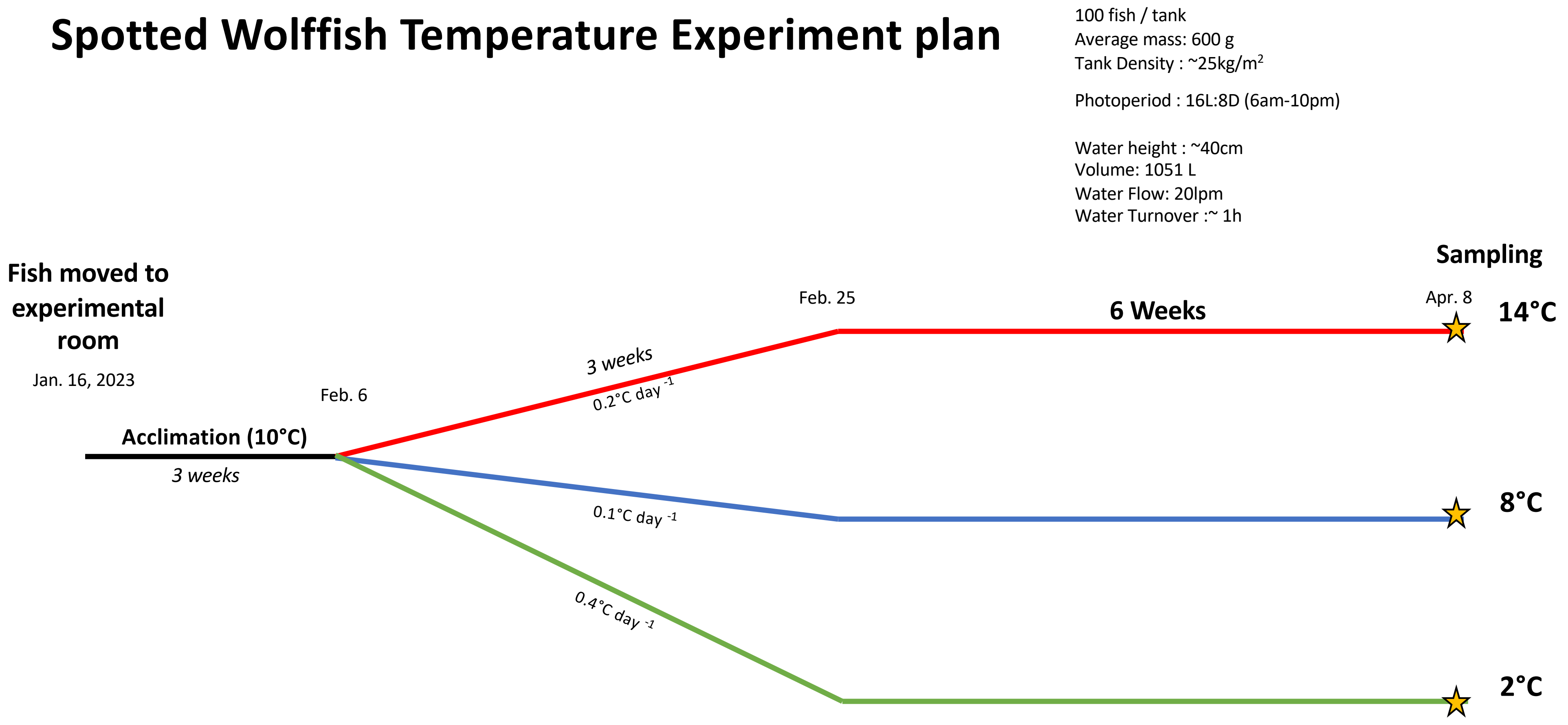

Supplemental Figure 2

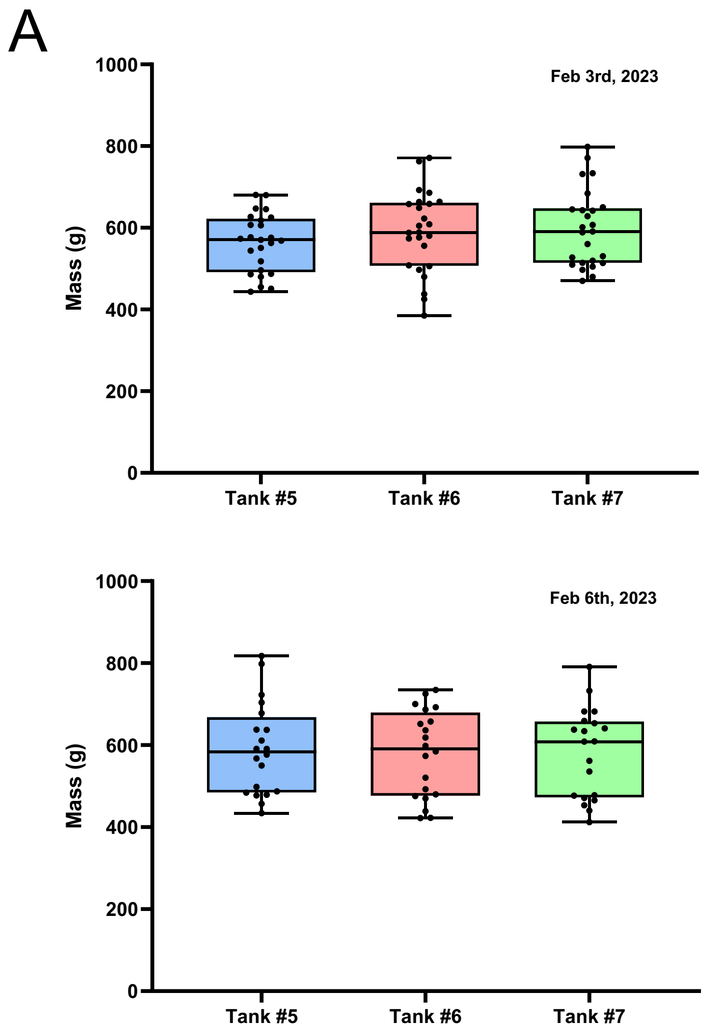

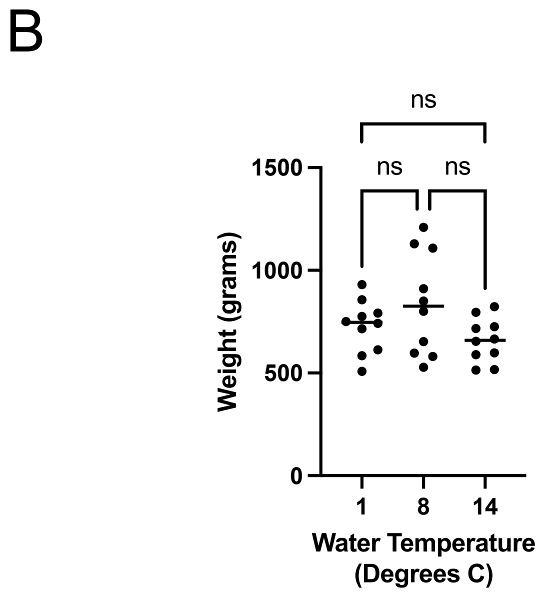

Supplemental Figure 2. Spotted wolffish mass during temperature experiment. A, Mass of randomly selected fish (Feb 3rd 2023 n=25 and Feb 6th 2023 n=20) in each tank prior to change in temperature. All fish were at 10°C, >100% air saturation. B, Mass of randomly sampled fish after acclimatization to each temperature and at experimental endpoint date of April 7th, 2023. 10 fish from each temperature were sampled for tissue harvesting and analysis. No statistically significant differences were found (Ordinary one-way ANOVA with multiple comparisons).

Supplemental Figure 3

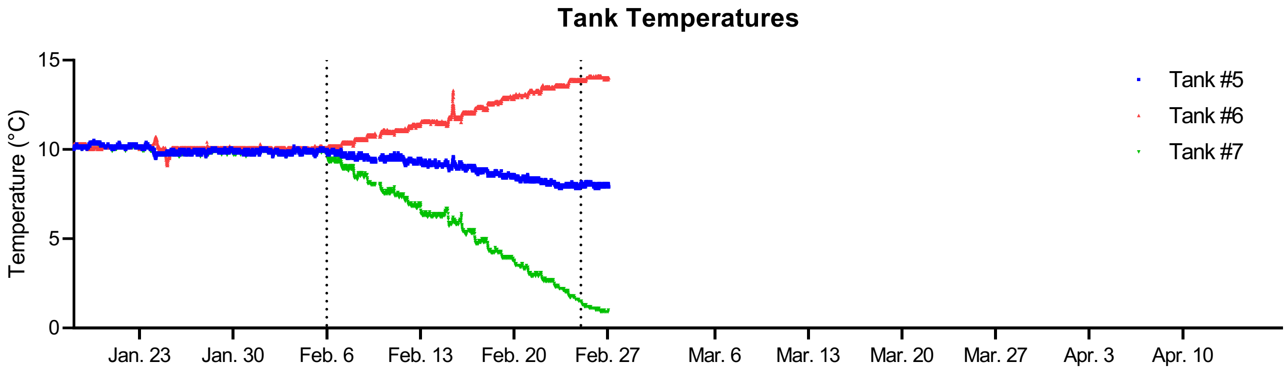

Temperature in tanks recorded every 10 minutes. Dotted lines indicate start and end of temperature changes (21 days).

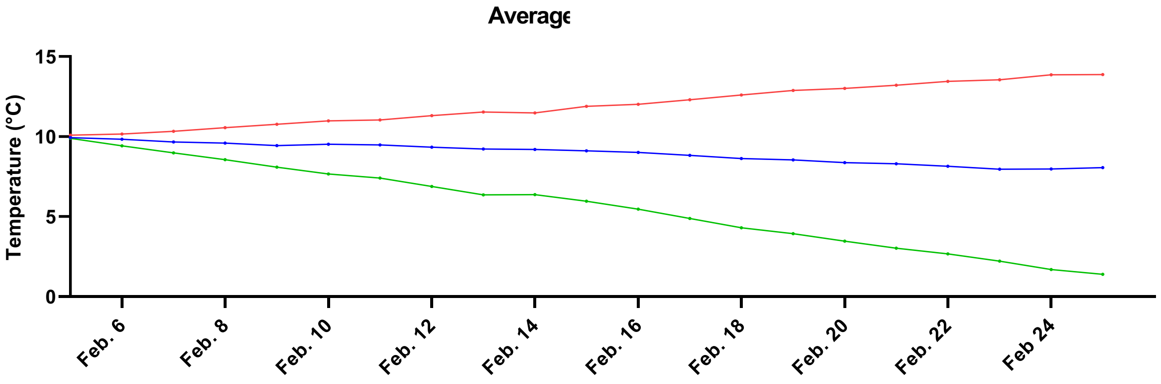

Average daily temperature in tanks over the duration of temperature adjustments (21 days).

Tank #5	Tank #6	Tank #7
Y = -0.1019*X + 10.07	Y = 0.2019*X + 9.721	Y = -0.4247*X + 10.31

Supplemental Figure 4

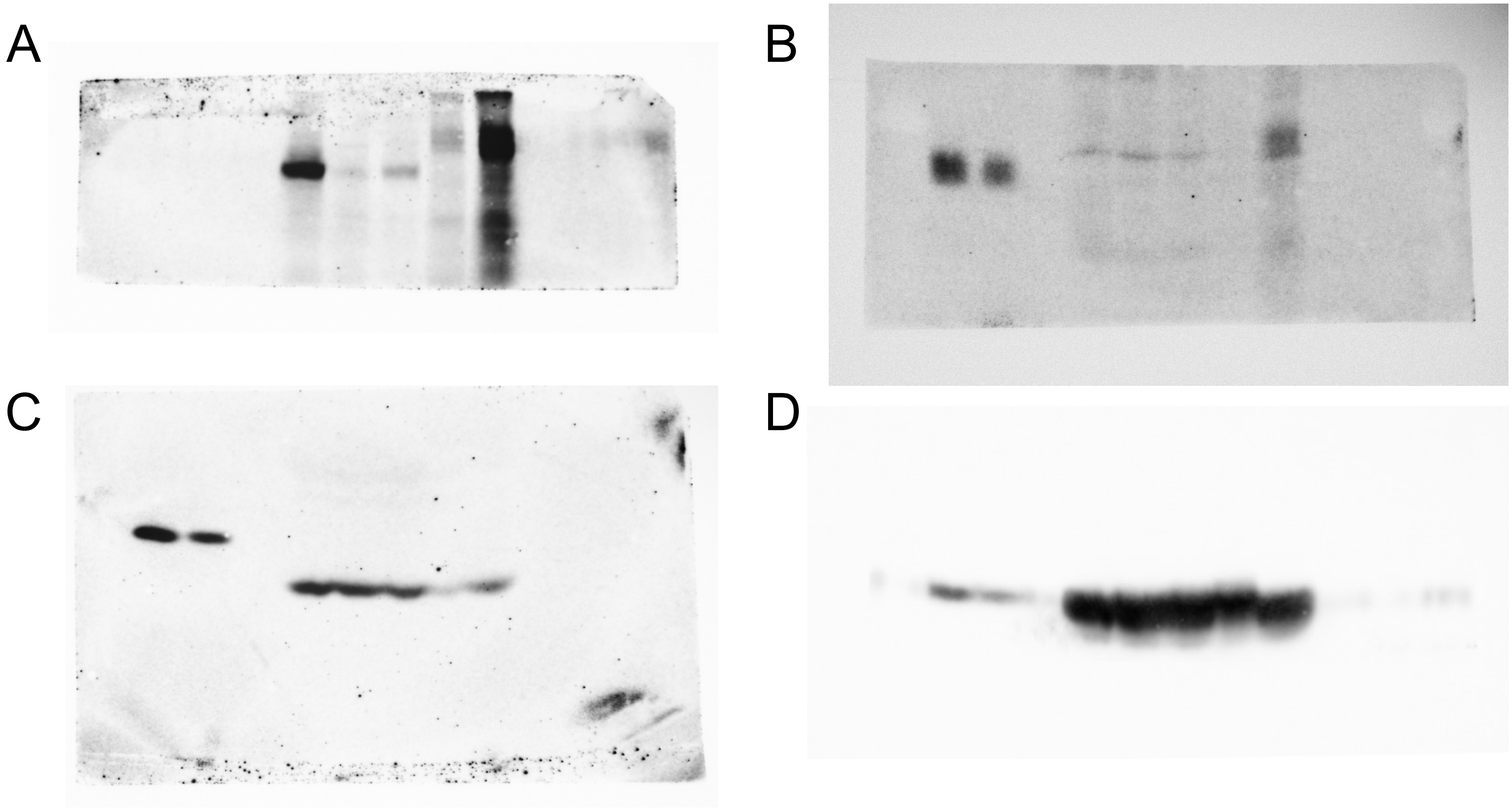

Supplemental Figure 4. Original, unprocessed version of western blots of spotted wolffish shown in manuscript Figure 4. A, Original, unprocessed version of western blot for CD45; B, Original, unprocessed version of western blot for Sox-9; C, Original, unprocessed version of western blot for PCNA; D, Original, unprocessed version of western blot for Tubulin.

Supplemental Figure 5

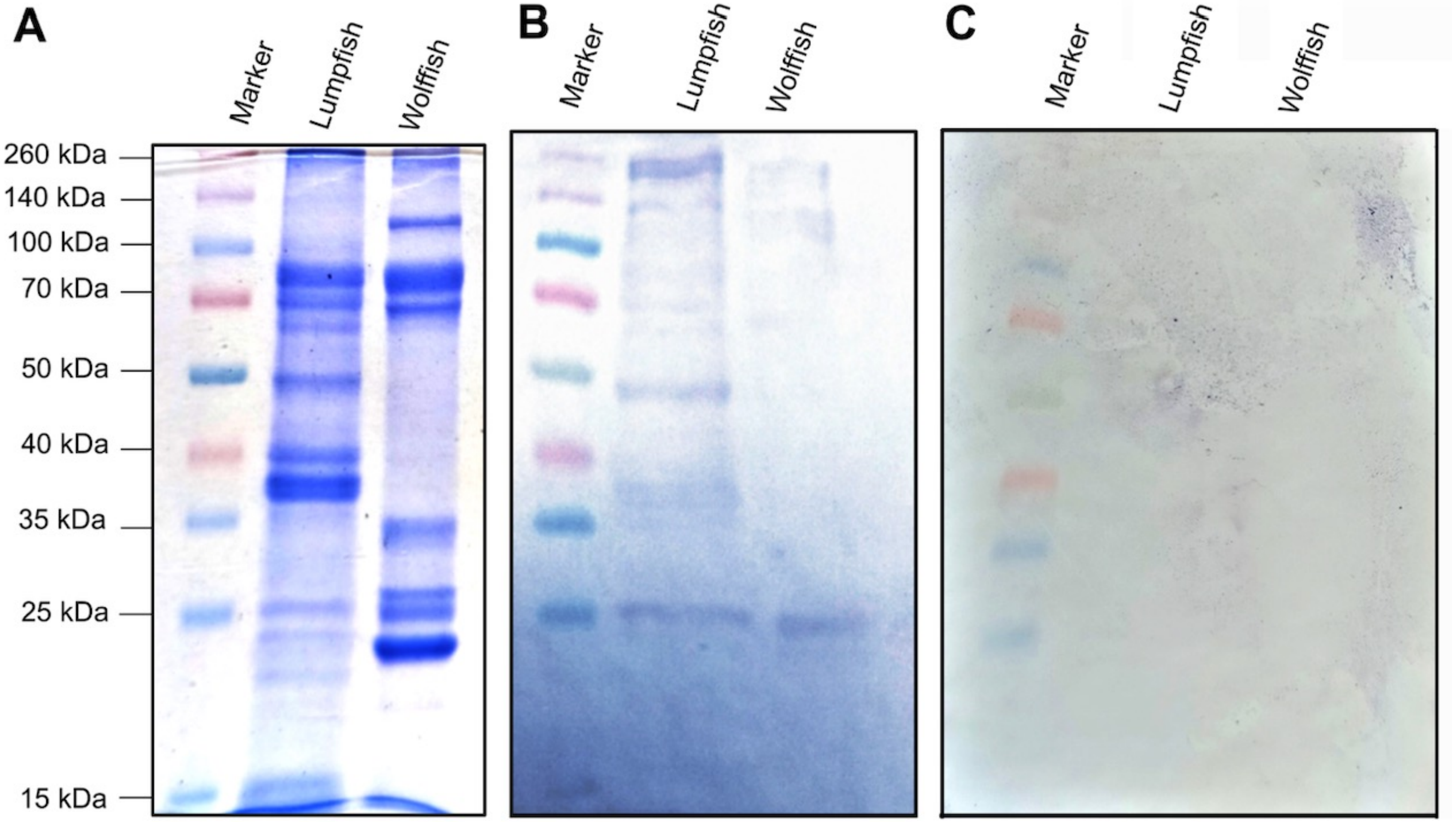

Supplemental Figure 5. Cross immune reaction of wolffish IgM and lumpfish IgM. A, SDS-PAGE of purified lumpfish and wolffish IgM stained with Coomassie Blue. **B,** Western blot of purified lumpfish and wolffish IgM using an anti-lumpfish IgM antibody. **C,** Western blot of purified lumpfish and wolffish IgM using a pre-absorbed mix of chicken IgY anti-lumpfish IgM antibody and wolffish IgM in place of the chicken IgY anti-lumpfish IgM alone.

Supplemental Figure 6 A, B

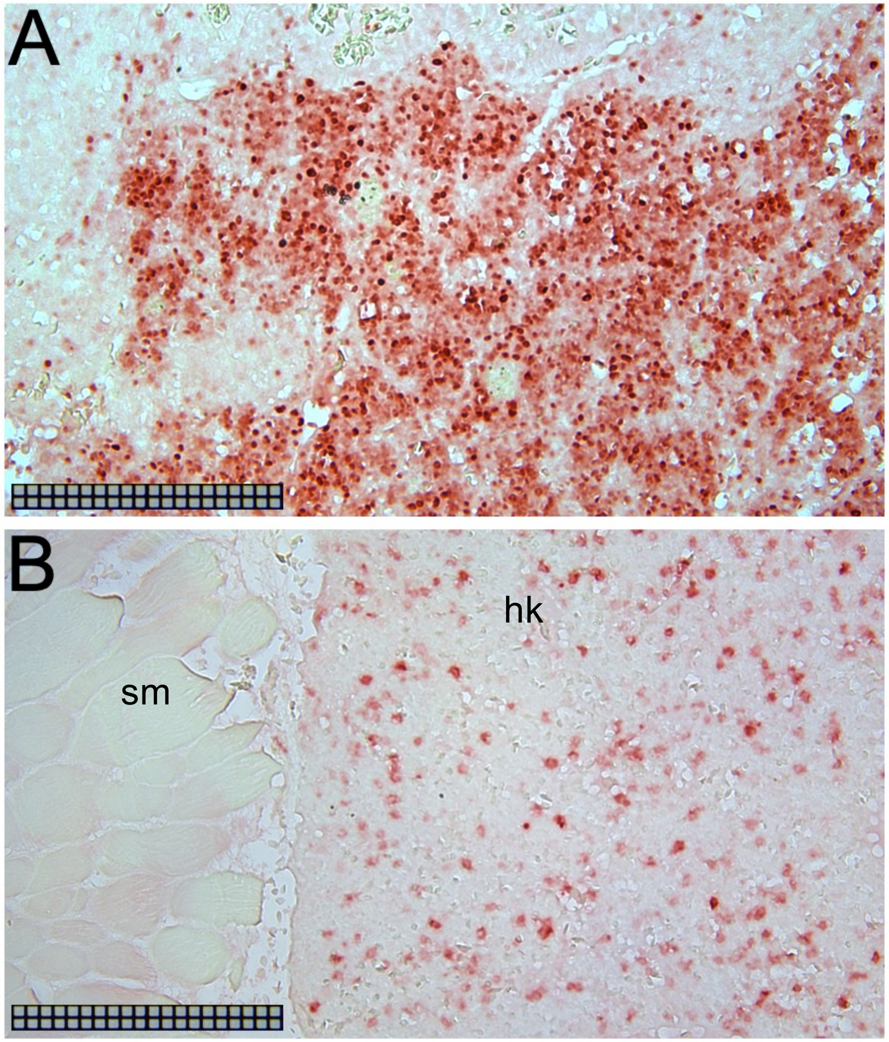

Supplemental Figure 6 C-F

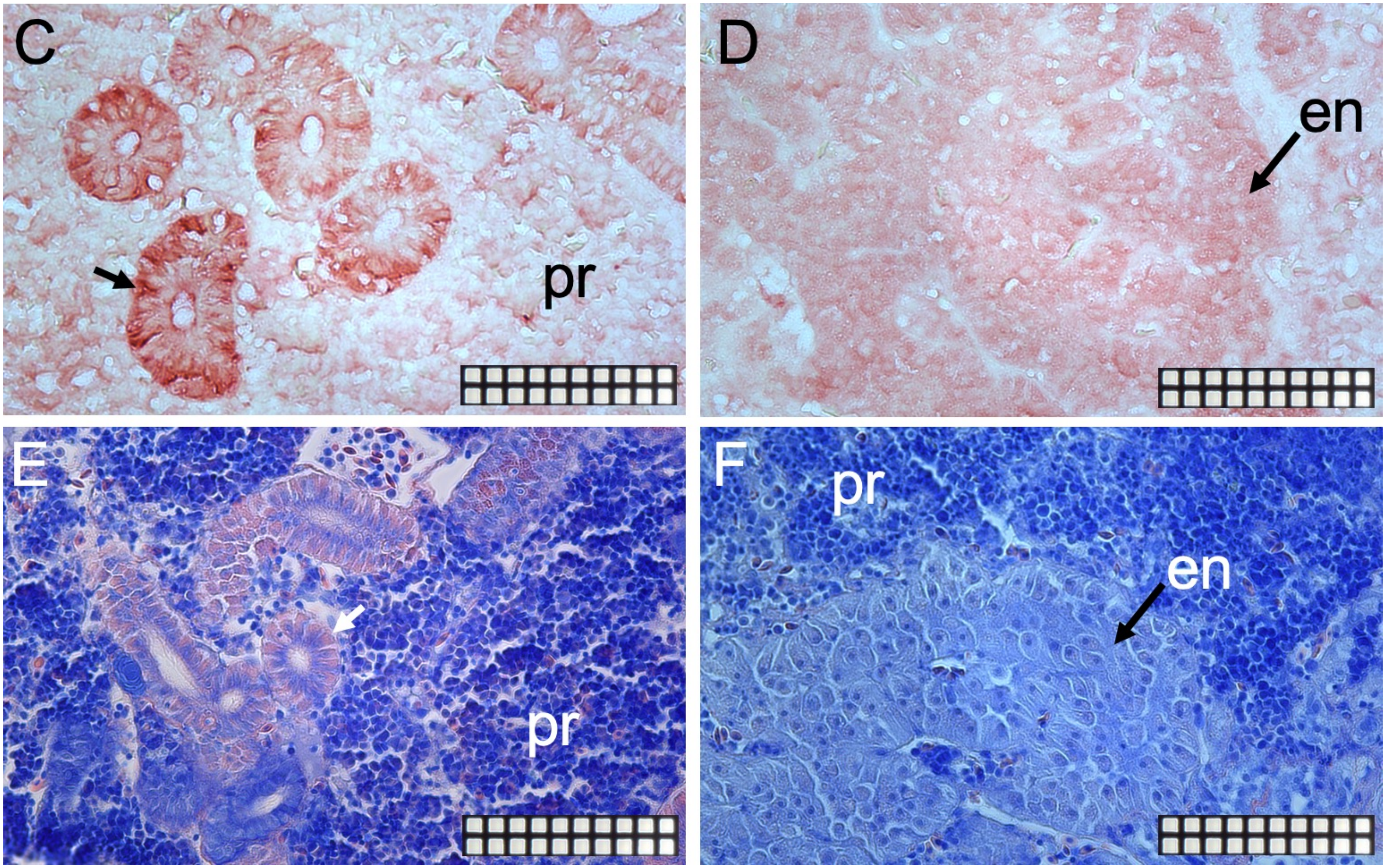

Supplemental Figure 6 G-I

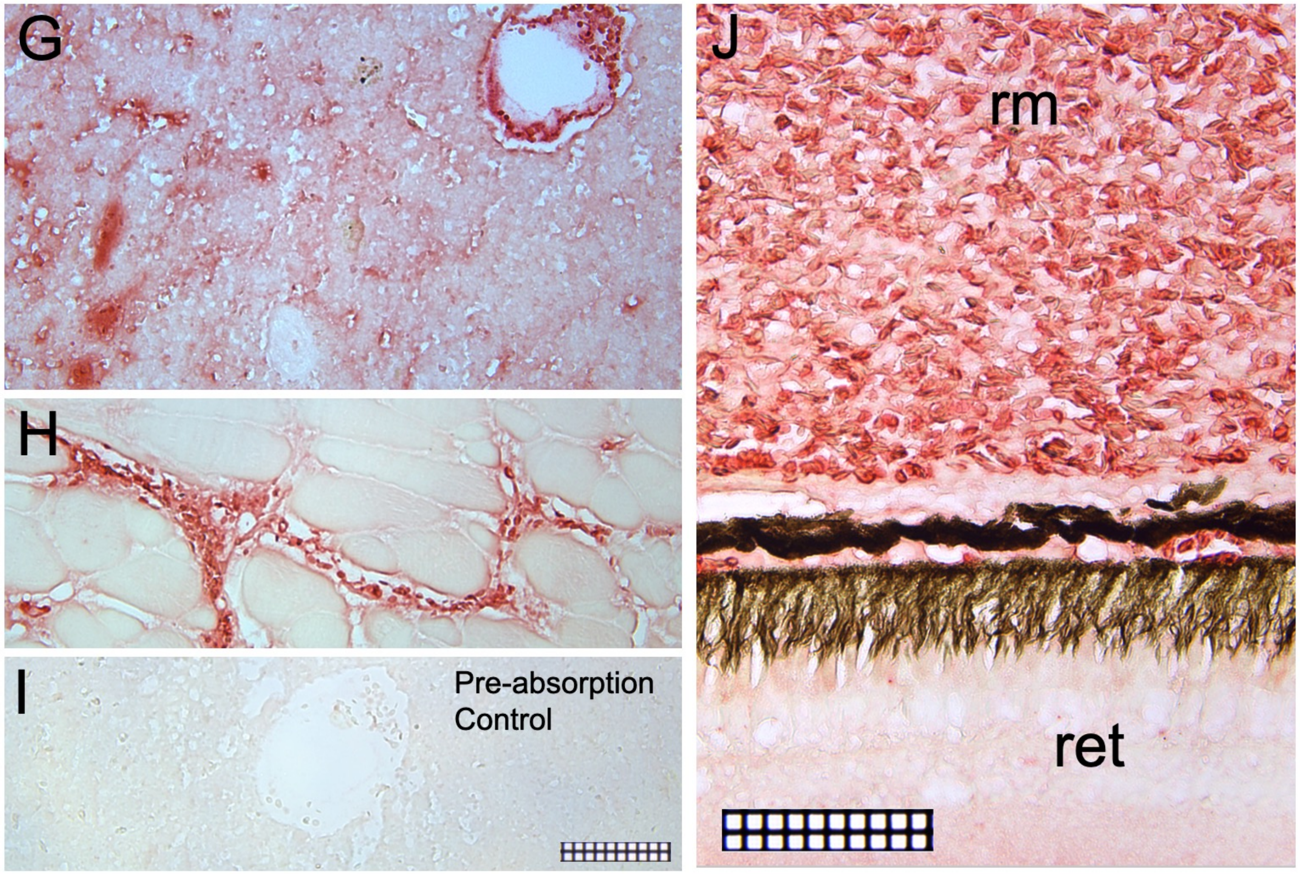

Supplemental Figure 6. Immunohistochemical validation of antibodies for PCNA, CD45, phosphotyrosine and IgM in spotted wolffish head kidney tissue. Expression of PCNA appeared as cell nuclear staining in head kidney parenchyma (A). Expression of CD45 appeared as a cellular membranous staining in head kidney (hk) parenchyma but not in adjacent sleletal muscle (sm) tissue (B). Expression of phosphotyrosine appeared as a cytoplasmic staining in head kidney renal tubules (small arrow in C), parenchyma (pr) and in endocrine (en) tissue (arrow in D). Geimsa stained of sections adjacent to C and D (E, F, respectively), further indicate the cellular histology. IgM was detected as a cellular and extracellular staining pattern in head kidney parenchyma (G) and, in cells and fluid in vasculature lumens (H). I, head kidney stained with purified-IgM-pre-absorbed anti-lumpfish IgM antibody. J, choroid body/rete mirabile stained for IgM. r, retina; rm, rete mirabile. All IHCs shown were performed using red alkaline phosphatase substrate. No counterstain was applied to the immunohistochemically stained sections in order to emphasize the IHC signals. Scale grid subdivisions are 10 micrometers. Representative images are shown.

Supplemental Figure 7

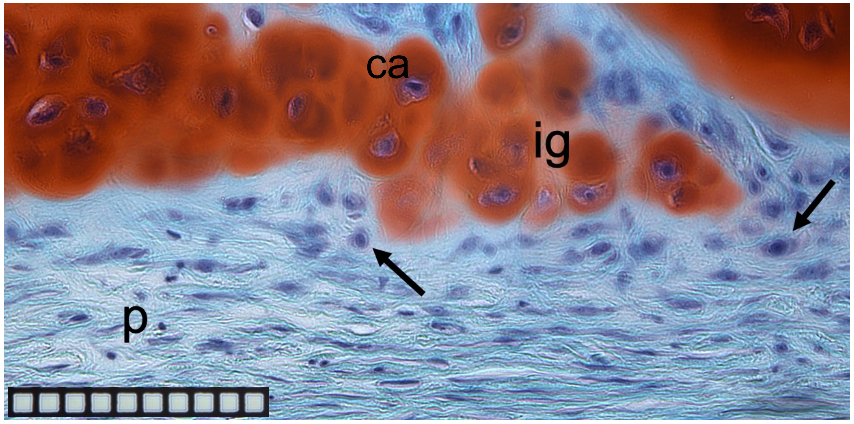

Supplemental Figure 7. A higher power view of Figure 5B displaying activated maturing chondrocytes (arrowed) separated from the cartilage (ca).

Supplemental Figure 8

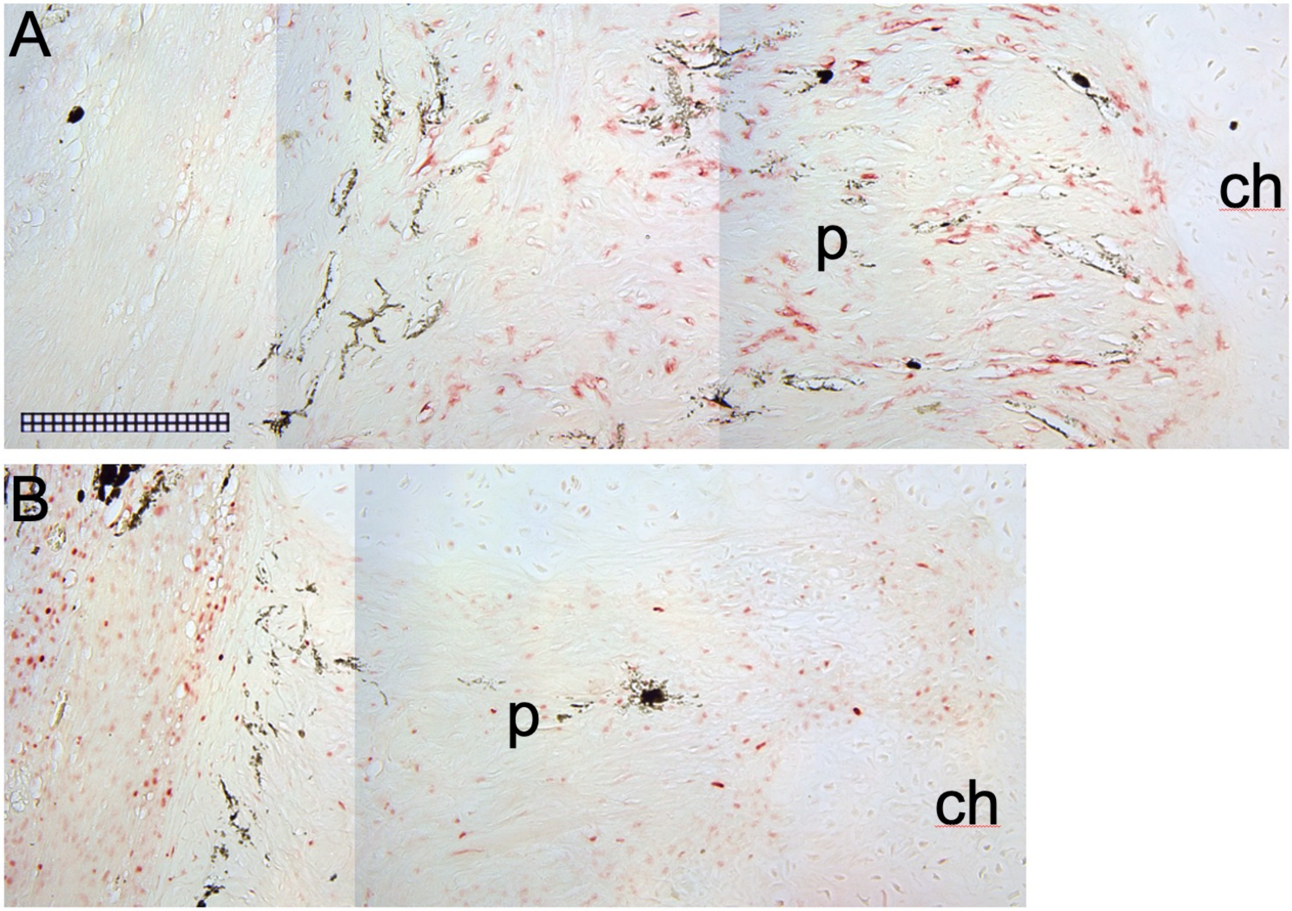

Supplemental Figure 8. **Immunohistochemical analysis of CD45 and PCNA in spotted wolffish scleral skeleton lesion-like tissue**. Broader scale views of the CD45 and PCNA stainings shown in Figure 6. Areas shown in A and B encompass the region indicated by the yellow box in Figure 2 (I). Staining for CD45 (A) appeared in cells of the perichondrium (p) and some areas consistent with the position of the maturing chondrocytess (ch). PCNA (B) appeared as a cellular nuclear staining in perichondrium (p),in some areas consistent with the position of the maturing chondrocytes(ch) and in connective tissues (ct). No counterstain was applied to the sections shown in order to emphasize the IHC signals. Cartilage, ca. Scale grid subdivisions are 10 micrometers.

Supplemental Figure 9

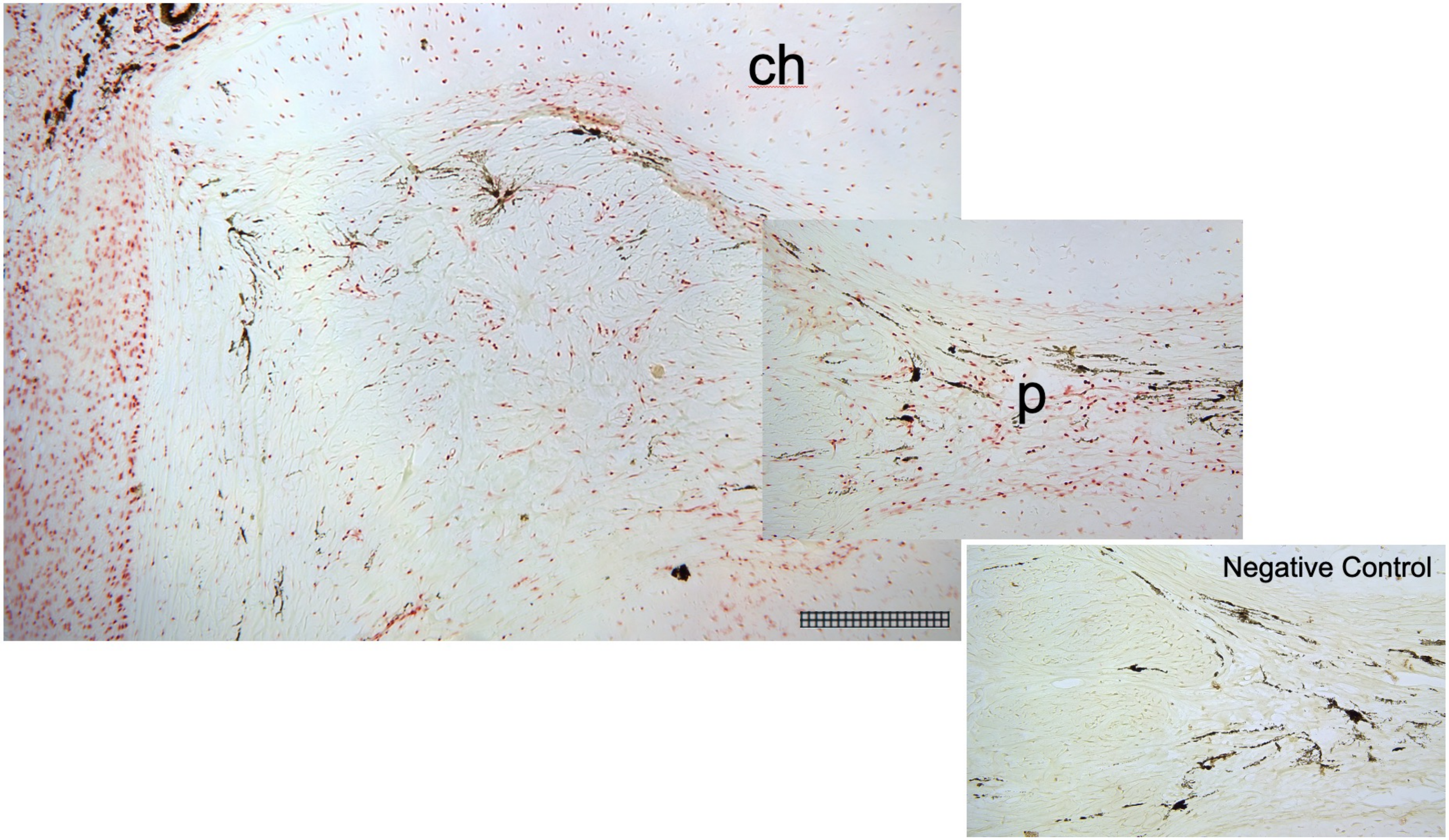

Supplemental Figure 9. **Immunohistochemical analysis of Sox9 in spotted wolffish scleral skeleton lesion-like tissue**. Top panels, broader scale views of Sox9 staining shown in Figure 6. Areas shown encompass and surround the yellow box region indicated in Figure 2 (I). Sox9 appeared as a cellular nuclear staining within the perichondrial (p) tracts projecting into the center of the lesion-like area and in the cartilage (ca). Sox9 was also expressed in areas of the maturing chondrocytes(ch) and in numerous cells within the connective tissue areas (ct). Negative control, no primary anti-Sox9 antibody. No counterstain was applied to the sections shown in order to emphasize the IHC signals. Scale grid subdivisions are 10 micrometers.

Supplemental Figure 10

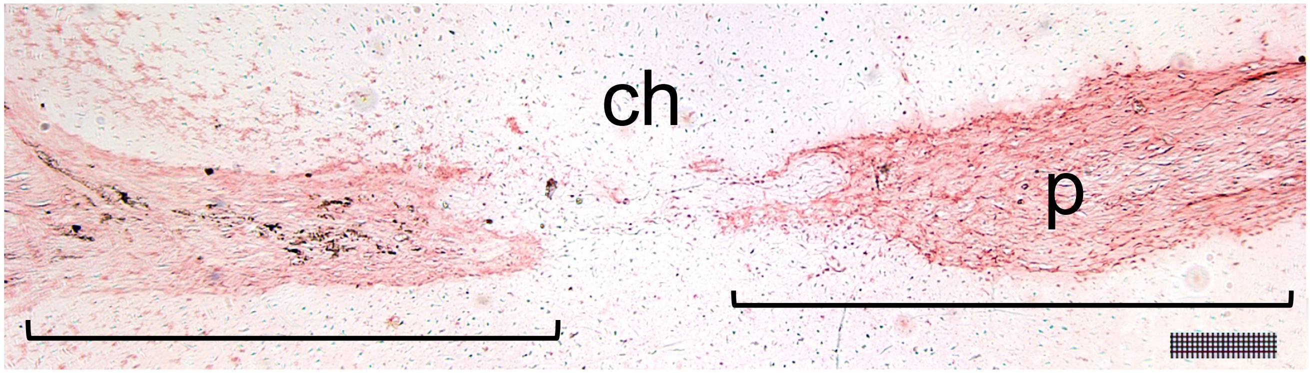

Supplemental Figure 10. **Immunohistochemical analysis of phosphotyrosine** i**n spotted wolffish scleral skeleton lesion-like tissue**. Broader scale views of the phosphotyrosine staining shown in Figure 6. Areas shown encompass the region indicated by the black box in Figure 2 (I). Staining for phosphotyrosine appeared in tracts in the perichondrium (p) projecting into both sides of the lesion-like tissue and areas consistent with the position of the maturing chondrocytes (ch). Brackets indicate broad areas of phosphotyrosine staining. No counterstain was applied to the section shown in order to emphasize the IHC signal. ca, cartilage. Scale grid subdivisions are 10 micrometers.

Supplemental Figure 11

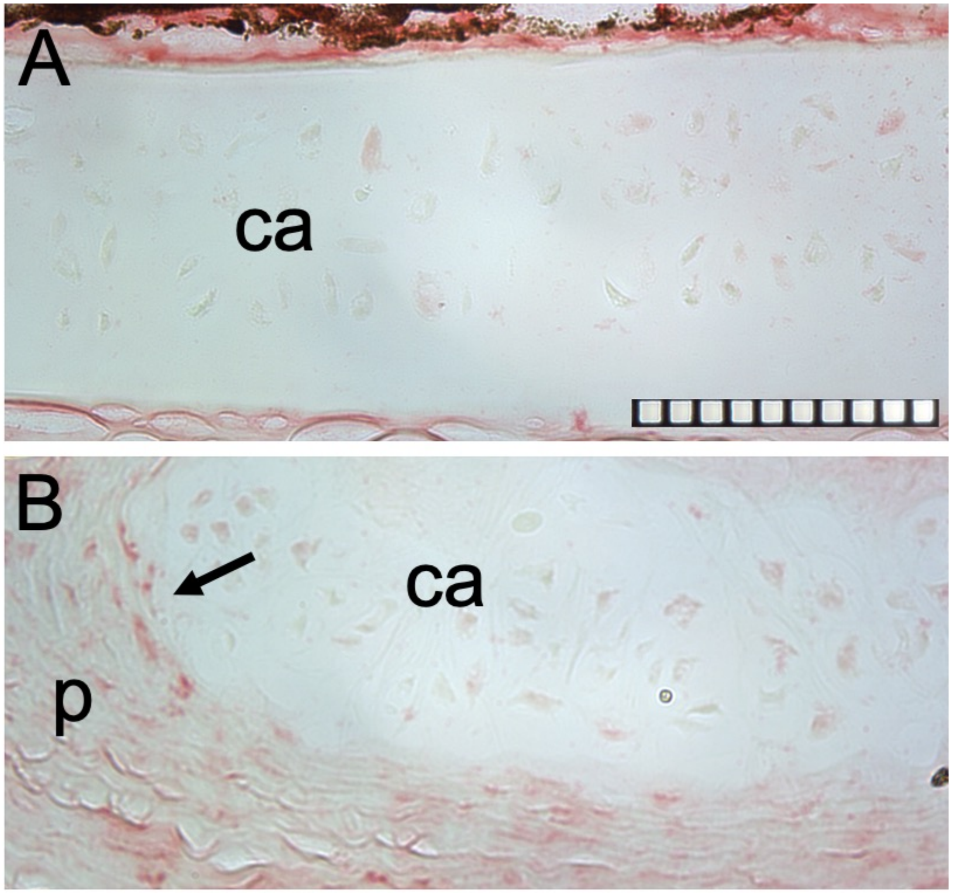

Supplemental Figure 11. **Immunohistochemical analysis of phosphotyrosine in normal spotted wolffish scleral cartilage**. (A, B) Phosphotyrosine IHC was performed on sections from normal spotted wolffish eyes. Tissue shown in (B) is located at edges of segments of scleral cartilage. Phosphotyrosine staining appeared in cells in the perichondrium (p) and in areas consistent with the position of the maturing chondrocytes (arrow). No counterstain was applied to the sections shown in order to emphasize the IHC signals. ca, cartilage. Scale grid subdivisions are 10 micrometers.
